# Unraveling verticillium wilt resistance: insight from the integration of transcriptome and metabolome in wild eggplant

**DOI:** 10.3389/fpls.2024.1378748

**Published:** 2024-05-28

**Authors:** Gengyun Li, Yunrong Mo, Junheng Lv, Shu Han, Wei Fan, Ying Zhou, Zhengan Yang, Minghua Deng, Bin Xu, Yanyan Wang, Kai Zhao

**Affiliations:** ^1^ Key Laboratory of Vegetable Biology of Yunnan Province, College of Landscape and Horticulture, Yunnan Agricultural University, Kunming, Yunnan, China; ^2^ College of Food Science and Technology, Yunnan Agricultural University, Kunming, China

**Keywords:** verticillium wilt, eggplant, transcriptome, metabolome, defense response

## Abstract

Verticillium wilt, caused by *Verticillium dahliae*, is a soil-borne disease affecting eggplant. Wild eggplant, recognized as an excellent disease-resistant resource against verticillium wilt, plays a pivotal role in grafting and breeding for disease resistance. However, the underlying resistance mechanisms of wild eggplant remain poorly understood. This study compared two wild eggplant varieties, LC-2 (high resistance) and LC-7 (sensitive) at the phenotypic, transcriptomic, and metabolomic levels to determine the molecular basis of their resistance to verticillium wilt. These two varieties exhibit substantial phenotypic differences in petal color, leaf spines, and fruit traits. Following inoculation with *V. dahliae*, LC-2 demonstrated significantly higher activities of polyphenol oxidase, superoxide dismutase, peroxidase, phenylalanine ammonia lyase, β-1,3 glucanase, and chitinase than did LC-7. RNA sequencing revealed 4,017 differentially expressed genes (DEGs), with a significant portion implicated in processes associated with disease resistance and growth. These processes encompassed defense responses, cell wall biogenesis, developmental processes, and biosynthesis of spermidine, cinnamic acid, and cutin. A gene co-expression analysis identified 13 transcription factors as hub genes in modules related to plant defense response. Some genes exhibited distinct expression patterns between LC-2 and LC-7, suggesting their crucial roles in responding to infection. Further, metabolome analysis identified 549 differentially accumulated metabolites (DAMs) between LC-2 and LC-7, primarily consisting of compounds such as flavonoids, phenolic acids, lipids, and other metabolites. Integrated transcriptome and metabolome analyses revealed the association of 35 gene–metabolite pairs in modules related to the plant defense response, highlighting the interconnected processes underlying the plant defense response. These findings characterize the molecular basis of LC-2 resistance to verticillium wilt and thus have potential value for future breeding of wilt-resistant eggplant varieties.

## Introduction

*Verticillium dahliae* (*V. dahliae*), a worldwide soil-borne fungus causing verticillium wilt, is one of the major diseases that harm agricultural production (Klosterman et al., 2009). Studies have shown that the pathogenic fungi overwinter through microsclerotia that are stress resistant, surviving in the soil for long periods and thus playing an important role in the disease cycle (Tzima et al., 2010). The hyphae enter host plants by formation of an infection structure that develops into conidia and expands upward from the roots during transpiration, entering the stems and leaves of plants and resulting in leaf curl, necrosis, defoliation, and discoloration, ultimately leading to plant withering and death (Sink and Grey, 1999). *Verticillium* wilt can occur throughout the entire growth period of eggplant, usually occurring during the fruiting period. In China 20%–40% of the eggplant fields are affected by *V. dahliae*, causing large-scale reductions in yield (Fradin and Thomma, 2006). *V. dahliae* contains multiple transposons and repetitive genes as well as genes involved in metabolic regulation and signal transmission (Zhang et al., 2020; Feng et al., 2023). This demonstration of genetic variation suggested that the mechanisms of pathogenicity may be diverse, and thus further analysis of disease resistance mechanisms is needed.

Researchers have isolated and cloned many genes related to the resistance to verticillium wilt from different plant species (Song et al., 2020). For example, the transient silencing of *GhNDR1* and *GhMKK2* by *Agrobacterium-*mediated VIGS in cotton (*Gossypium hirsutum*) led to increased sensitivity of cotton to *V. dahliae*, while silencing of *GhNPR1* had no significant effect on antagonism toward the pathogen (Gao et al., 2011). In addition, *GbNRX1* can regulate cotton resistance by clearing extracellular ROS (Li et al., 2016). *VdMKK1* is significantly upregulated during the invasion of host cotton by *V. dahliae*, and it plays a crucial role in the invasion process by regulating the expression of cell wall synthesis genes and thereby maintaining the integrity of cell walls (Li et al., 2023). *GbHyPRP1* negatively regulates the resistance of cotton plants to *V. dahliae* by regulating cell wall structures and ROS levels (Yang et al., 2018). In addition to defense-related proteins, further studies have been conducted on the role of transcription factors in *Verticillium* resistance (Song et al., 2020). A full-length cDNA library and expressed sequence tag (EST) sequencing in cotton inoculated with *V. dahliae* screened two ethylene-related genes, GbERF1 and GbERF2; overexpression of GbERF2 induced the expression of related genes and PR proteins by regulating the ethylene pathway and enhancing the resistance of tobacco (Zuo et al., 2007). GhWRKY1 enhanced resistance to verticillium wilt by increasing lignification (Hu et al., 2021). VdMRTF1 can mediate cotton resistance to *V. dahliae* by regulating various processes, including melanin biosynthesis, development of microsclerotia, and resistance to elevated Ca^2+^ levels (Lai et al., 2022). An NAC transcription factor, GhATAF1, and GhMYB108 were both induced by *V. dahliae* infection and promoted defense responses (Cheng et al., 2016; He et al., 2016). Some oxidoreductases and hydrolases, including peroxidase and cell wall proteolytic enzymes, play important roles in the resistance response to verticillium wilt. Several studies of cotton resistance to verticillium wilt have isolated these protease genes (Zuo et al., 2005; Xu et al., 2011a), and related studies have shown that after inoculation with pathogens, the peroxidase activity of resistant varieties is significantly higher than that of susceptible varieties (Gayoso et al., 2010; Xu et al., 2011a). In addition, hydrolytic enzymes such as chitinase and glucanase are involved in the resistance to verticillium wilt by degrading fungal cell walls (Dubery and Slater, 1997).

The mechanism of resistance to verticillium wilt has been extensively studied in the Solanaceae (Kawchuk et al., 2001; Kruijt et al., 2005). The *Ve* genes, including *Ve1* and *Ve2*, were early discovered genes for resistance to verticillium wilt. The genes encode leucine-rich repetitive (LRR) proteins (Kruijt et al., 2005). When these two *Ve* genes were expressed separately in susceptible potato varieties, both produced resistance to isolated strains of *V. dahliae* (Kawchuk et al., 2001). Similar resistance mechanisms have also been found in homologous gene phenotypes of *Ve* in other Solanaceae crops, including tomato (Chai et al., 2003) and eggplant (Liu et al., 2012). Jue et al. (2014) cloned two genes conferring strong resistance to verticillium wilt, *StoNPR1* and *StoWRKY6*, from wild eggplant *Torubam*. The genes enhanced the resistance of eggplant to verticillium wilt by synthesizing proteins that mediated the salicylic acid signaling pathway. The interaction between miR482e and nucleotide binding sites (NBS) and leucine-rich repeat (LRR) motifs in potatoes led to increased severity of *V. dahliae* infection (Yang et al., 2015).

Although various genes have been identified in the resistance system to verticillium wilt, little is known about the complex molecular mechanisms underlying defense responses in eggplant. In recent years, the rise of genome, transcriptome, metabolome, and proteome analyses has resulted in an in-depth understanding of the resistance mechanisms toward verticillium wilt (Zhang et al., 2020; Feng et al., 2023). Xu et al. (2011a) identified 3,442 defense-responsive genes from the transcriptomic profiles of *V. dahliae*-infected cotton using RNA-seq technology. Further research on these differentially expressed genes revealed the key role of lignin metabolism in the resistance of cotton to verticillium wilt (Xu et al., 2011b). A comprehensive analysis utilizing transcriptome and metabolome revealed the intrinsic components and DEGs in *Arabidopsis* inoculated with *V. dahliae* (Su et al., 2018). In eggplant, Tomah et al. (2023) identified genes encoding enzymes involved in microsclerotia degradation based on the analysis of transcriptomics. Differentially expressed genes between the *V. dahliae* infected and uninfected samples identified 10 different gene network modules and 22 hub genes with potential roles in regulating cotton defense against *V. dahliae* infection (Xiong et al., 2021).

To better understand the molecular networks of plant–pathogen interactions in eggplant, we investigated two wild eggplant varieties with different resistance phenotypes, LC-2 (high resistance) and LC-7 (sensitive) and analyzed the differences in enzyme activity associated with resistance. We identified transcriptome changes occurring during the process of *V. dahliae* infection in order to characterize the biological processes involved based on Gene Ontology (GO) terms of the differentially expressed genes using RNA-seq technology. A co-expression analysis identified key hub genes involved in regulating the resistance pathways of eggplant to verticillium wilt. Additionally, a metabolomic analysis identified key differential metabolites. A correlation analysis between transcriptome and metabolome was used to explore the relationship between differentially expressed genes and metabolic components, thereby providing a theoretical basis for the in-depth study of the mechanism of action of eggplant against verticillium wilt.

## Materials and methods

### Plant material and *Verticillium dahliae* infection treatment

Eggplant (*Solanum melongena* L.) local varieties LC-2 and LC-7 were collected from Langcang County, Pu’er City, Yunnan Province. *V. dahliae* was provided by the Institute of Vegetables and Flowers, Chinese Academy of Agricultural Sciences. Seeds of each variety were soaked in 600 mg·L^-1^ gibberellic acid for germination and sown in a plug containing a substrate (perlite:peat soil = 1:2 V/V). The plug was placed in a constant temperature incubator with a temperature of 30°C during the day and 22°C at night, with a light/dark cycle of 14 h/10 h. After 5–6 true leaves had grown, the *V. dahliae* was artificially inoculated at a concentration of 1×10^8^ cfu·mL^-1^ using the root–injury–root immersion and root irrigation methods. The fibrous roots of the seedlings were cut off at 0.2 cm, immersed in the *V. dahliae* solution for 20 min, and planted in plastic pots. Each pot was transferred back to the constant temperature incubator under the same conditions as before and irrigated with 20 mL *V. dahliae* solution every two days. Uninoculated individuals were used as controls, the leaves of three plants were randomly selected and sampled, each plant was taken as a biological replicate. In the treatment group, two time points were selected at 20 days and 40 days after inoculation, three biological replicates were set at each inoculation time point for LC-2 and LC-7, with one biological replicate investigating 20 plants.

### Identification of the disease resistance level

The classification of disease level was as follows: Level 0: asymptomatic; Level 1: less than 25% of the leaves turning yellow; Level 2: 25%–50% of the leaves turning yellow; Level 3: more than 50% of the leaves turning yellow with some diseased leaves fall off; Level 4: the leaves of the whole plant are yellow, and even the whole plant dies.

Disease index (DI) = (disease level × number of plants in this disease level)/(highest disease level × number of investigated plants)×100

High resistance (HR): DI = 0; Resistance (R): 0 < DI ≤ 20; Moderate Resistance (MR): 20 < DI ≤ 40; Susceptibility (S): 40 < DI ≤ 60; High susceptibility (HS): DI>60.

### Measurement of enzyme activities

The third and fourth leaves from the top were selected as experimental materials. Experiments were conducted with three biological replicates by spectrophotometry. The kits were purchased from Beijing Boxbio Science & Technology Co., Ltd., and the determination of each index was performed according to the manufacturer’s instructions. A total of six physiological indicators were measured, including polyphenol oxidase (PPO, kit code: AKAO004C), superoxide dismutase (SOD, kit code: AKAO001C), peroxidase (POD, kit code: AKAO005C), phenylalanine ammonia lyase (PAL, kit code: AKAM012U), β-1,3 glucanase (GUN, kit code: AKSU038C), and chitinase (CHT, kit code: AKSU045C). The activities were normalized by the weight of leaf. The change of light absorption value of A410nm of the reaction system by 0.005 per minute per g tissue was defined as a unit of enzyme activity of PPO. When the inhibition rate of xanthine oxidase coupling reaction system in each g tissue was 50%, the activity of SOD in the reaction system was defined as an enzyme activity unit at 560 nm. The change of A470nm per minute by 0.01 per g tissue per mL system was one unit of enzyme activity of POD. One enzyme activity of PAL was defined as a 0.1 change in absorbance value at 290 nm per minute per g of tissue per mL of the reaction system unit. The production of 1 mg of reducing sugar per g of tissue sample per hour was defined as a unit of enzyme activity of GUN at 540 nm. The production of 1 μg of N-acetylglucosamine per g of tissue per hour was defined as a unit of enzyme activity of CHT at 585 nm.

### Metabolomic analysis

The 18 LC-2 and LC-7 leaf samples (two varieties × three biological replicates × three time points) of treatment and control groups at 0, 20, and 40 days were freeze-dried and crushed into powder using a mixer mill (MM 400, Retsch). Three biological replicates were set for each sample. Metabolite profiling was performed using a widely targeted metabolome method by Wuhan Metware Biotechnology Co., Ltd. (Wuhan, China) (http://www.metware.cn/). The samples were obtained from a total of 100 mg of powder extracted at 4°C overnight with 1.0 ml of 70% aqueous methanol and then centrifuged at 10,000 × g for 10 min. The extracts were detected by UPLC-ESI-MS/MS (UPLC: SHIMADZU Nexera X2; MS: Applied Biosystems 4500 QTRAP). The following method was employed for metabolite quantification and analysis. Scans using both Linear ion trap (LIT) and triple quadrupole (QQQ) were acquired on a triple quadrupole-linear ion trap mass spectrometer (API 4500 Q TRAP LC/MS/MS System) equipped with an ESI Turbo Ion-Spray interface. Multiple-reaction monitoring (MRM) was utilized in conjunction with a self-compiled database (Metware database, MWDB) to perform metabolite quantification. Differences between LC-2 and LC-7 samples at the same time point were analyzed using orthogonal partial least-squares discrimination analysis (OPLS-DA) and variable importance in projection (VIP). Identification of differential metabolites was performed within and between the local varieties. Within each local variety, samples from the 0-day of each variety (representing the initial state) were used as controls to identify differentially accumulated metabolites (DAMs) at 20 and 40 days. Between local varieties, LC-7 was used as a control to identify DAMs between LC-2 and LC-7 at the same treatment time point. Metabolites with significant differences in content were set with thresholds of variable importance in projection (VIP) ≥ 1 and fold change ≥ 2 or ≤ 0.5 (Guo et al., 2022). The Kyoto Encyclopedia of Genes and Genomes (KEGG) compound database (http://www.kegg.jp/kegg/compound/) was utilized for metabolite annotation.

### Transcriptome sequencing and data analysis

Transcriptome sequencing was performed by using the leaf samples as for the metabolomic analysis, and three biological replicates in each sample were set. Total RNA was extracted from the samples using Trizol reagent (Invitrogen, USA), and RNA quality was assessed through 1% agarose gel electrophoresis. A NanoDrop 2000 (NanoDrop Technologies, Wilmington, DE, United States) and an Agilent 2100 Bioanalyzer (Agilent Technologies, USA) were used to determine the RNA concentration and purity, respectively. High-quality RNAs were then used for RNA sequencing by Wuhan Metware Biotechnology Co., Ltd. (Wuhan, China) (http://www.metware.cn/) using the Illumina HiSeq platform (Illumina Inc., San Diego, CA, USA). After a standard library construction process (Li et al., 2022a), clean reads were aligned to the eggplant reference genome (http://eggplant-hq.cn/Eggplant/home/index) using the Hisat2 program. The mapped reads of each sample were assembled by StringTie, and featureCounts v1.5.0-p3 was used to count the number of reads mapped to each gene to calculate FPKM. Differentially expressed genes (DEGs) were identified within and between local varieties by using the same comparison strategy as used in the DAMs identification. DESeq2 software was used to identify the differentially expressed genes using the criteria absolute value of the log2 fold change ≥ 1 and false discovery rate (FDR) < 0.05. The Gene ontology enrichment for DEGs was visualized using WEGO (https://wego.genomics.cn/) and REVIGO (http://revigo.irb.hr/). The Kyoto Encyclopedia of Genes and Genomes (KEGG) database (https://www.genome.jp/kegg/) was used to analyze the pathways of DEGs.

### Gene co-expression network analysis

A gene co-expression network was constructed by using the WGCNA R package. The FPKM values of DEGs were normalized by using the limma R package. According to the results of multiple soft thresholding powers calculation (**Supplementary Figure S1**), the network was constructed by setting a power of 18, a minModuleSize of 30, and a mergeCutHeight of 0.25. Module eigengenes (MEs) were defined as the first principal component of the expression matrix of the corresponding module and were calculated by using the moduleEigengenes function with default parameters for each module. After network construction, the top 30% of weighted edges were analyzed, as millions of edges were generated. The module hub genes were identified as the nodes with the top 10% degree in each module.

### Correlation analysis of metabolome and transcriptome data

The potential associations between the transcriptome and metabolome data were identified by assessing the correlations between metabolite content and the expression pattern of each gene and the ME of each gene co-expression module. Briefly, Pearson’s correlation coefficient (PCC) for the content of DAMs and the expression of DEGs was calculated using the “cor” function in R, and significant correlations were screened using a criterion of an absolute value greater than 0.80 and a p-value less than 0.05. The PCC was also calculated between metabolite content and ME using the “moduleEigengenes” function in the WGCNA R package. Considering the capacity of ME to partially represent the average expression of module genes, it is important to note that the average per se may mask some of the correlations between genes and metabolites. Therefore, a lower criterion than DAMs-DEGs correlation was used, with absolute coefficients greater than 0.6 and p-values less than 0.05 considered as indicating a significant correlation between metabolites and gene modules. Finally, metabolites that demonstrated significant correlations with both the modules and the genes within the modules were selectively filtered, thereby establishing a comprehensive association between the metabolome and transcriptome.

### Quantitative real-time PCR (qRT-PCR) validation

To validate gene expression patterns discovered by transcriptome sequencing, 13 transcription factors were randomly selected, and their expression patterns were validated by qRT-PCR. Total RNA of each LC-2 and LC-7 sample was reverse transcribed into cDNA using a PrimeScriptTM RT reagent kit (Takara, Dalian, China). The qRT-PCR reaction was performed using TB GreenTM Premix Ex TaqTM II (Takara, Dalian, China) on a Roche LightCycler 96 system (Roche) following the manufacturers’ instructions. Relative quantitative analysis of data was performed by the 2^−ΔΔCT^ method, with GAPDH as the reference gene (Zhao et al., 2013; Zhou et al., 2014). The primers used for RT-qPCR are listed in **Supplementary Table S1**. Three independent biological samples of eggplant plants of LC-2 and LC-7 under the same treatment were used, which were different from the transcriptome samples.

## Results

### LC-2 exhibits greater resistance and physiology shifts in response to *V. dahliae* than LC-7

**Figure 1:**
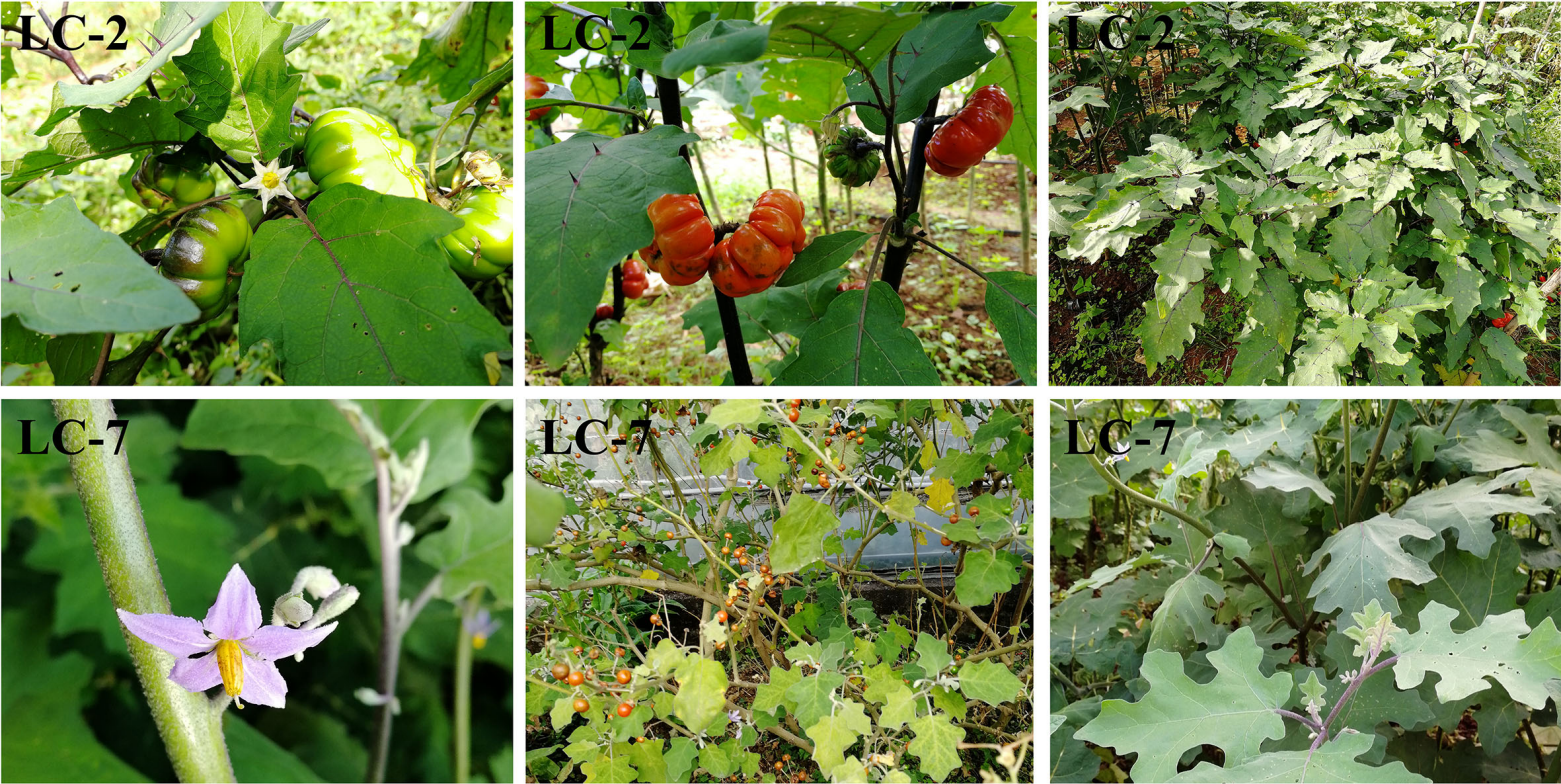
Performance of typical agronomic traits of LC-2 and LC-7 in the field, including petal color, leaf spines, and fruit traits.

LC-2 and LC-7 were grown in a facility greenhouse to ensure consistent environmental conditions. Agronomic traits were investigated when the respective first panicle fruits were ripe. LC-22 and LC-7 showed significant differences in main stem color, main stem prickle, leaf spines, corolla color, single fruit weight, fruit shape, and fruit surface furrows (**Figure 1**; **Supplementary Table S2**).

**Figure 2:**
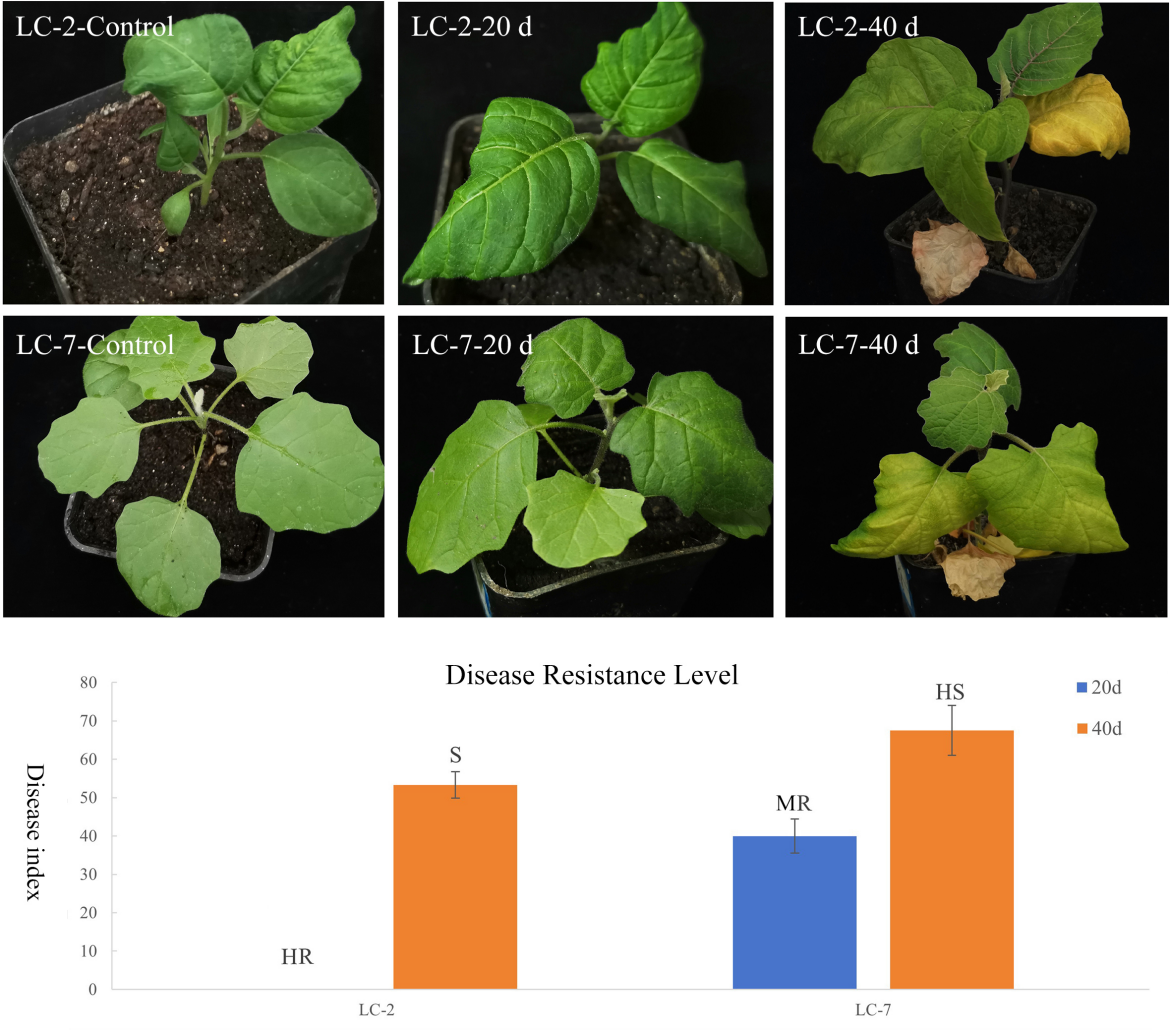
Performance and resistance levels of LC-2 and LC-7 inoculated with *V. dahliae.* Control: uninoculated, 20 d: 20 d after inoculation, 40 d: 40 d after inoculation., HR, High Resistance; R, Resistance; MR, Moderate Resistance; S, Susceptibility; HS, High susceptibility. The results are means of three biological replicates ± standard deviation (SD).

To clarify the differences in resistance between LC-2 and LC-7, the resistance level was identified at 20 and 40 days after *V. dahliae* inoculation. The results showed that the overall resistance of LC-2 was stronger than that of LC-7. Specifically, 20 days after inoculation, LC-2 showed high resistance and LC-7 showed moderate resistance to *V. dahliae*, and 40 days after inoculation, LC-2 showed susceptibility, while LC-7 showed high susceptibility (**Figure 2**).

To analyze the characteristics of changes in the enzymes related to disease resistance in LC-2 and LC-7, the six indices PPO, SOD, POD, PAL, GUN, and CHT were measured in leaves at 20 and 40 days after *V. dahliae* inoculation. The results showed that the six activities demonstrated gradually increasing trends with time after inoculation with *V. dahliae.* The values of the six indexes in LC-2 were higher than those in LC-7 under normal growth conditions. All six indices were positively correlated with resistance to *V. dahliae* (**Table 1**).

**Table 1:** Changes in enzyme activities associated with disease resistance at different times (20 d and 40 d) after inoculation of *V. dahliae*.

	PPO (U/G)		SOD (U/G)		POD (U/G)		PAL (U/G)		GUN (U/G)		CHT (U/G)	
**0**	7.8±0.5	18.6±1.0^**^	189.8±8.9	219.8±7.9^**^	813.7±30.3	2100.7±89.5^**^	10.7±0.9	16.7±0.8^**^	0.3±0.01	1.5±0.06^**^	12.4±0.3	16.2±1.2^**^
**20D**	19.8±1.3	38.9±2.5^**^	486.5±20.3	533.4±10.4^**^	1235.6±45.7	3809.8±30.9^**^	40.7±3.2	58.9±3.2^**^	2.4±0.1	4.8±0.2^**^	12.8±0.9	20.3±1.5^**^
**40D**	78.9±5.3	90.7±5.6	768.9±10.5	812.7±20.4^**^	1567.8±56.3	7698.5±90.6^**^	70.8±4.5	112.3±7.8^**^	5.1±0.2	6.9±0.3^**^	14.2±1.2	32.3±2.3^**^

The yellow background represents LC-2. The results are means of three biological replicates ± standard deviation (SD) (Student’s t-test, *P < 0.05, **P < 0.01).

### The DAMs of LC-2 and LC-7 in response to *V. dahliae* infection

**Figure 3:**
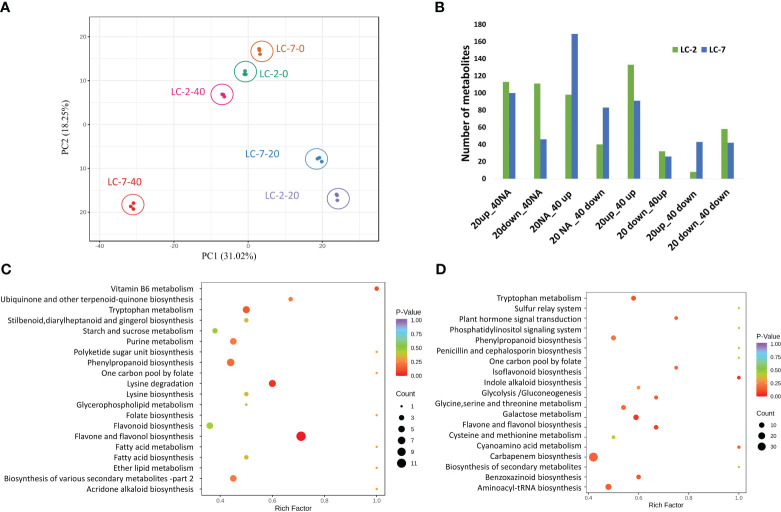
Summary of the metabolomes of LC-2 and LC-7. **(A)** Principal component analysis (PCA) of LC-2 and LC-7 samples. **(B)** The numbers of differentially accumulated metabolites (DAMs) in LC-2 and LC-7. **(C, D)** The statistics of KEGG enrichment of DAMs of LC-2 vs. LC-7 at 20 days **(C)** and 40 days **(D)**.

To gain a better understanding of the differences in metabolites between LC-2 and LC-7 in response to *V. dahliae* infection, metabolic profiling was conducted using samples from the treatment and control groups of LC-2 and LC-7 at 0, 20, and 40 days. A total of 840 metabolites were detected; these included flavonoids (175), phenolic acids (142), lipids (130), alkaloids (85), amino acids and derivatives (60), organic acids (58), nucleotides and derivatives (38), terpenoids (31), lignans and coumarins (27), steroids (16), quinones (3), and other metabolites (75). A principal component analysis (PCA) revealed that the three biological replicates of each sample tended to group together. The 0 d and 20 d samples had a closer distance between cultivars in the 2D PCA analysis, while the 40 d sample had a greater distance between LC-2 and LC-7 (**Figure 3A**).

Within each variety, differential accumulation of metabolites was compared between 20-day and 0-day samples as well as between 40-day and 0-day time points. Totals of 593 and 600 DAMs were identified in LC-2 and LC-7, respectively (**Supplementary Table S3**). Based on the differential expression patterns at the two time points, we classified the expression patterns of the DAMs into eight categories: 20up_40NA, 20down_40NA, 20NA_40up, 20NA_40down, 20up_40up, 20down_40up, 20up_40down, and 20down_40down (“Up,” “down,” and “NA” represent upregulation, downregulation, and no differential expression at that time point, respectively). The results showed that LC-2 had the most 20up_40up-type metabolites, followed by 20up_40NA-type metabolites (**Figure 3B**), indicating that many metabolites in LC-2 responded to *V. dahliae* infection and increased in content at 20 days. In contrast, LC-7 had the largest number of differential metabolites in the 20NA_40 up category (**Figure 3B**), suggesting that metabolites in LC-7 responded to *V. dahliae* infection and began to accumulate at 40 days. A comparison of LC-2 and LC-7 identified 549 DAMs between local varieties. A total of 346 DAMs (176 upregulated and 170 downregulated) were identified at the 20-day time point, with the most common metabolites being flavonoids (96), phenolic acids (63), and lipids (49) (**Supplementary Figure S2A**). These DAMs were significantly enriched in pathways such as flavone and flavonol biosynthesis, lysine degradation, and tryptophan metabolism (**Figure 3C**). At the 40-day time point, there were 399 DAMs (180 upregulated and 219 downregulated) between LC-2 and LC-7, with the most common being phenolic acids (85), flavonoids (77), and lipids (65) (**Supplementary Figure S2B**). These DAMs were significantly enriched in pathways such as biosynthesis of secondary metabolites, flavone and flavonol biosynthesis, and cysteine and methionine metabolism (**Figure 3D**).

### Transcriptomic signatures of LC-2 and LC-7 in response to *V. dahliae* infection

After raw data filtering and examination of the sequencing error rate and the distribution of GC content, an average of 6.74 Gb of clean data was generated for each library. The average error rate was 0.03; the average Q30 was 92.59%, and the GC content ranged from 42.13% to 46.54% (**Supplementary Table S4**). The difference in genetic background led to distinct average mapping rates for LC-2 and LC-7: LC-2 achieved 81.28%, while LC-7 attained 70.26%, with fluctuations within 3% for each library. The lower mapping rate in LC-7 is not attributed to contamination by *V. dahliae*, as samples without infection also displayed mapping rates similar to other samples. For the sequences mapped to the reference genome, 85.42%–89.78% of the clean reads were mapped to exon regions, and approximately 3.90%–6.31% of the clean reads were mapped to intron regions (**Supplementary Table S5**). Finally, 36568 transcripts were identified.

**Figure 4:**
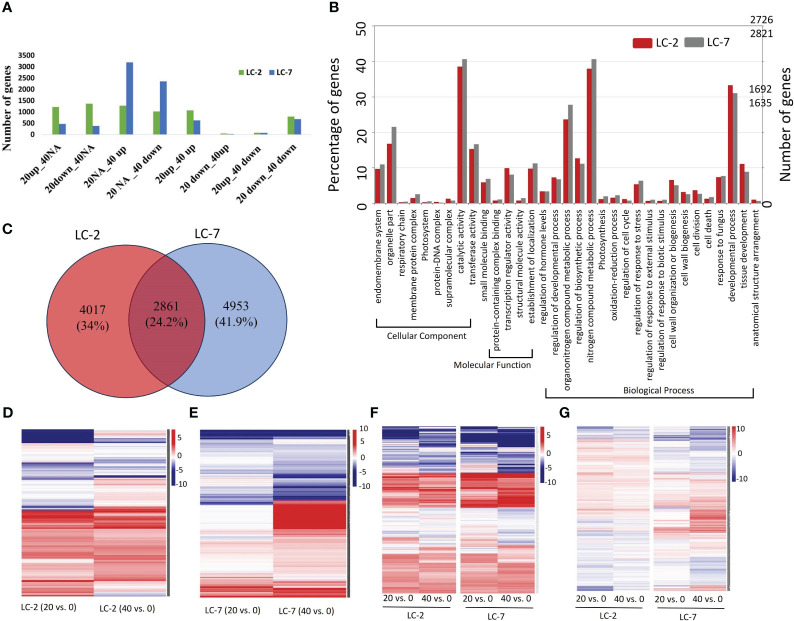
The expression and functional characteristics of *V. dahliae*-responsive genes in LC-2 and LC-7. **(A)** The numbers of differentially expressed genes (DEGs) in LC-2 and LC-7. **(B)** The GO enrichment results and corresponding numbers of genes for DEGs in LC-2 and LC-7. **(C)** Comparison and distribution of DEGs in 20-day and 40-day samples of LC-2 and LC-7, using 0-day samples as a control. **(D–G)** Expression profiles of 4017 LC-2-specific DEGs **(D)**, 4953 LC-7-specific DEGs **(E)**, 351 DEGs with similar expression patterns between LC-2 and LC-7 **(F)**, and 2861 DEGs with different expression patterns between LC-2 and LC-7 **(G)**. The color of the heatmap represents the log2 fold change of the comparison between pairs. Red and blue respectively represent positive and negative fold change values, with higher saturation indicating higher absolute values.

The temporal expression pattern of each gene was determined by comparing 20-day vs. 0-day and 40-day vs. 0-day levels within each variety. Using the criteria for identifying DEGs, 6,878 and 7,814 DEGs were identified in LC-2 and LC-7, respectively. The DEGs in LC-2 were predominantly distributed among the 20down_40NA, 20up_40NA, 20NA_40up, and 20up_40up categories, with a similar number of DEGs in each category (**Figure 4A**). In contrast, the DEGs in LC-7 were largely distributed in the 20NA_40up and 20NA_40down categories (**Figure 4A**). These findings suggest that the response of LC-7 to *V. dahliae* infection occurred later than that of LC-2.

The comparative analysis of biological processes associated with DEGs revealed significant differences between LC-2 and LC-7. The percentage of genes enriched in developmental processes, cell wall organization or biogenesis, and regulation of biosynthetic processes was substantially higher in LC-2 than in LC-7. Conversely, the percentage of genes enriched in processes related to response to fungus was similar between the two varieties (**Figure 4B**).

The comparison of DEGs between LC-2 and LC-7 revealed that LC-2 and LC-7 had 4,017 and 4,953 unique DEGs, respectively (**Figure 4C**). These genes exhibited variety-specific differential expression patterns (**Figures 4D, E**). Among these, a total of 2,861 DEGs were common to both LC-2 and LC-7 (**Figure 4C**). Further analysis of their expression patterns at the same time points between varieties (LC-2 vs. LC-7) revealed that 351 genes did not meet the criteria for differential expression between varieties and exhibited similar expression patterns over time (**Figure 4F**). The remaining 2,510 genes showed completely different expression patterns between LC-2 and LC-7 (**Figure 4G**).

### Diverse types of DEGs were involved in various biological processes

**Figure 5:**
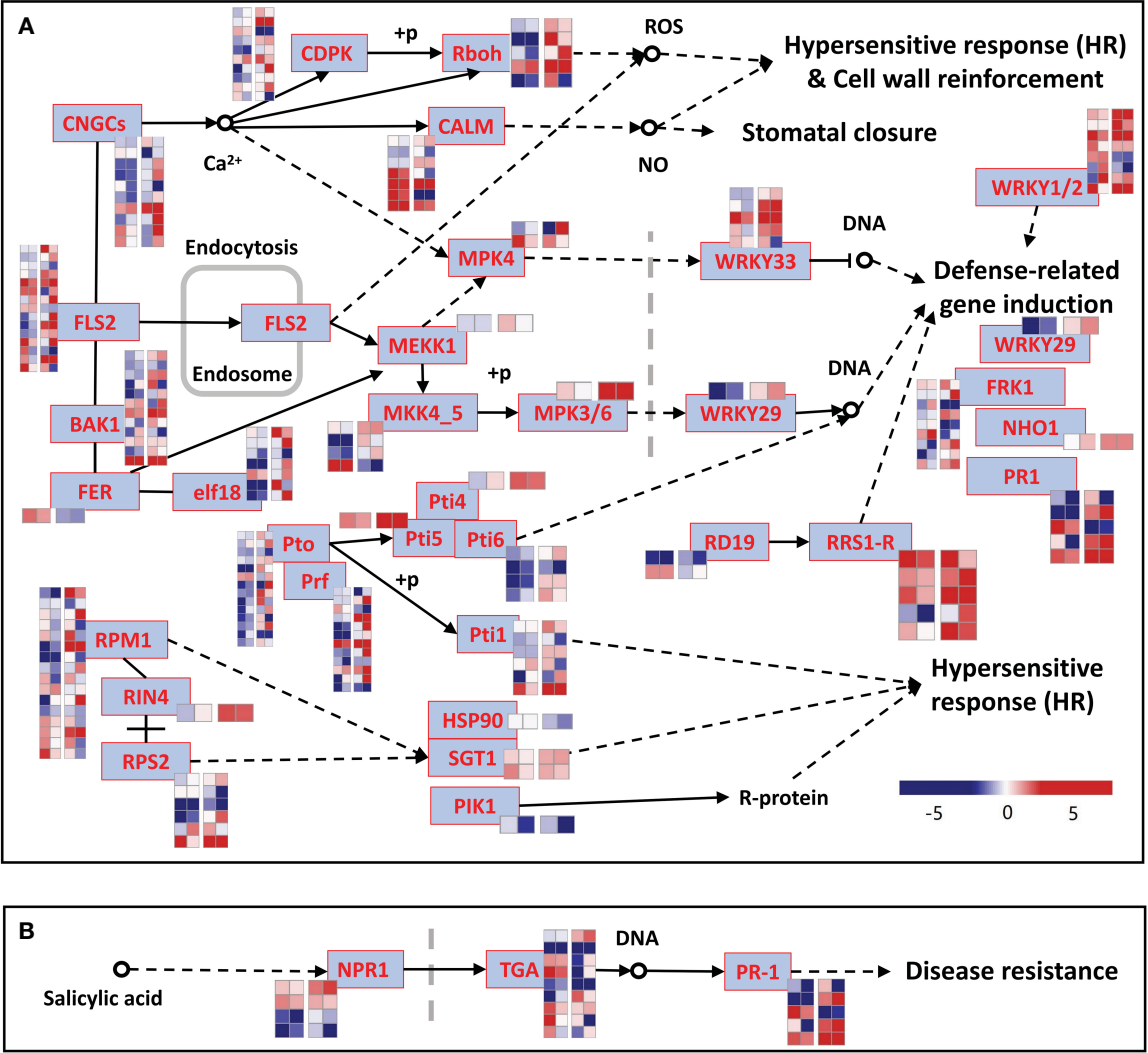
DEGs in KEGG pathways related to the pathogen defense response. **(A)** Plant–pathogen interaction (ko04626). **(B)** Disease resistance-related pathways in plant hormone signal transduction (ko04075). The blue rectangles represent genes, and the heatmap beside each gene shows the log2 fold change of 20- and 40-day samples for LC-2 and LC-7, respectively. The 0-day samples of LC-2 and LC-7 were used as controls to calculate the fold change for each variety.

The DEGs of each type were subjected to Gene Ontology (GO) enrichment analysis. The analysis showed that among the 4017 LC-2-specific DEGs, the genes were involved in defense response, biological synthesis processes (such as cell wall biogenesis, spermidine biosynthetic process, cinnamic acid biosynthetic process, and regulation of cutin biosynthetic process), and developmental processes (**Supplementary Figure S3A**). In contrast, the 4953 LC-7-specific DEGs were primarily involved in defense response to other organisms, plant hormone biosynthetic process, and metabolic processes (such as abscisic acid and salicylic acid) (**Supplementary Figure S3B**). The 351 DEGs with similar expression patterns between varieties were involved in defense response, plant hormone response processes (such as salicylic acid and abscisic acid), and compound biosynthesis processes (**Supplementary Figure S3C**). Moreover, the 2510 genes that were shared between varieties but with different expression patterns were involved in defense response and antibiotic metabolic and response processes as well as other processes such as sterol biosynthetic process and cell wall organization (**Supplementary Figure S3D**). Moreover, a total of 408 DEGs were involved in the plant–pathogen interaction pathway (ko04626, **Figure 5A**) and the disease resistance-related pathway in plant hormone signal transduction (ko04075, **Figure 5B**). Most of these genes in these two pathways showed different expression patterns between LC-2 and LC-7 (**Figures 5A, B**). **Supplementary Table S6** provides detailed information on these genes.

### Gene co-expression modules related to defense response against *V. dahliae* infection

**Figure 6:**
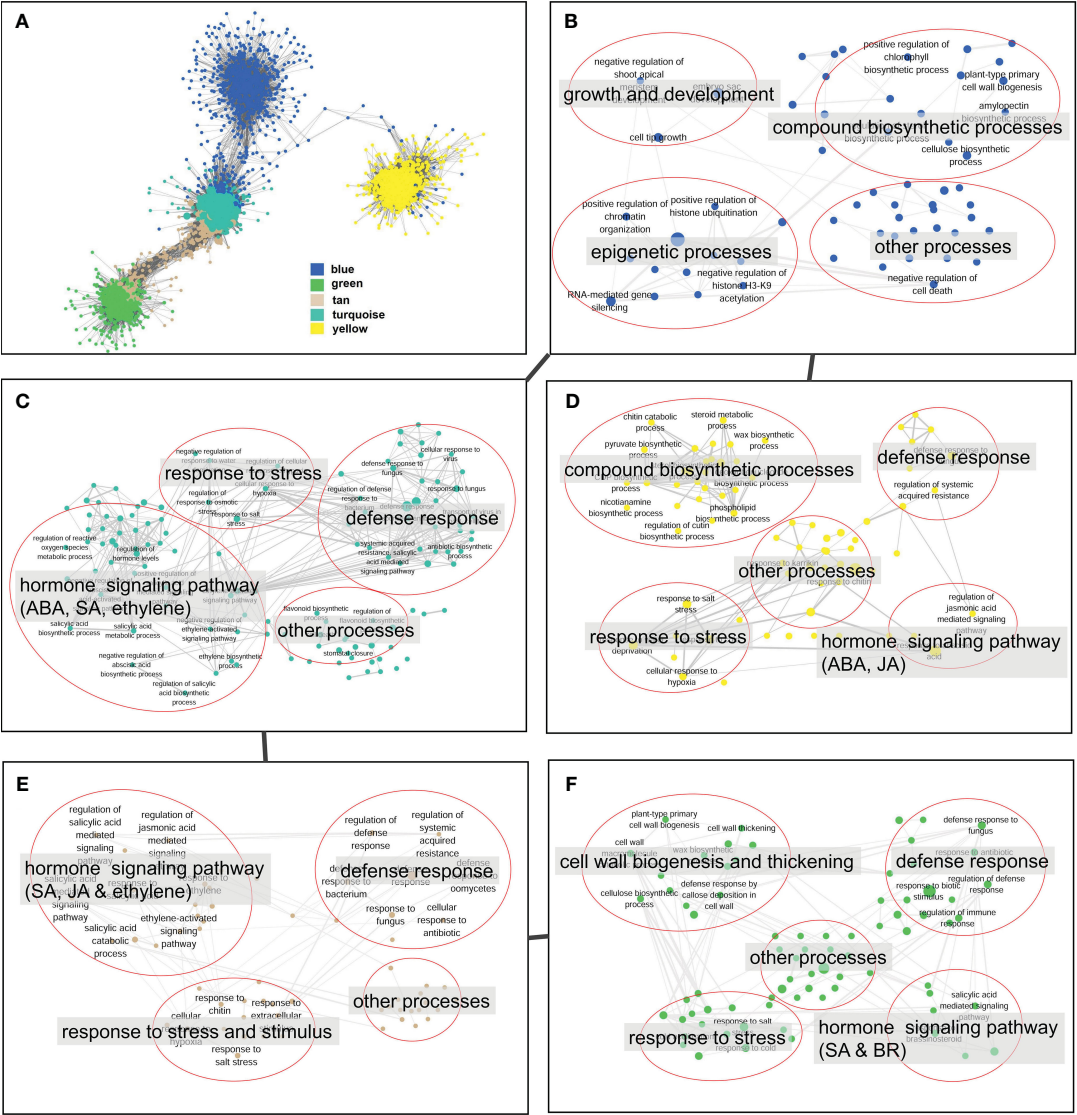
Topological structure and functional relevance of gene co-expression modules. **(A)** Gene co-expression sub-network constructed by five key modules (blue, green, tan, turquoise and yellow). Modules are represented by node genes with different colors. **(B–F)** Biological processes of blue **(B)**, turquoise **(C)**, yellow **(D)**, tan **(E)**, and green **(F)** modules. Biological processes are represented by nodes with module colors, and biological processes with similar functions are categorized in red circles and summarized by tags.

The co-expression network analysis resulted in the identification of 29 modules that encompassed a total of 6,258 nodes. To confirm the genes associated with defense response, we analyzed the distribution of genes related to the plant–pathogen interaction pathway (ko04626) and the disease resistance-related pathway in plant hormone signal transduction (ko04075) across different modules. The findings revealed that 72% of the disease resistance-related genes were recruited in the turquoise, green, tan, blue, and yellow modules, which collectively accounted for 49.1% of the total nodes in the network. Thus, these five modules were considered to be associated with the defense response against *V. dahliae* infection. A closer examination of the network’s topological structure revealed that the blue, turquoise, tan, and green modules exhibited a transitional connection structure, and the blue module was linked to the yellow module through several co-expression genes (**Figure 6A**). The functional analysis demonstrated that the genes in the blue module were primarily involved in biological processes such as growth and development, epigenetic regulation, and compound biosynthetic processes (**Figure 6B**). In contrast, the turquoise, tan, green, and yellow modules were enriched with genes related to defense response, hormone signaling pathway, and stress response processes (**Figures 6C–F**). The green module was notably enriched with a large number of genes related to cell wall biogenesis and thickening (**Figure 6F**).

**Figure 7:**
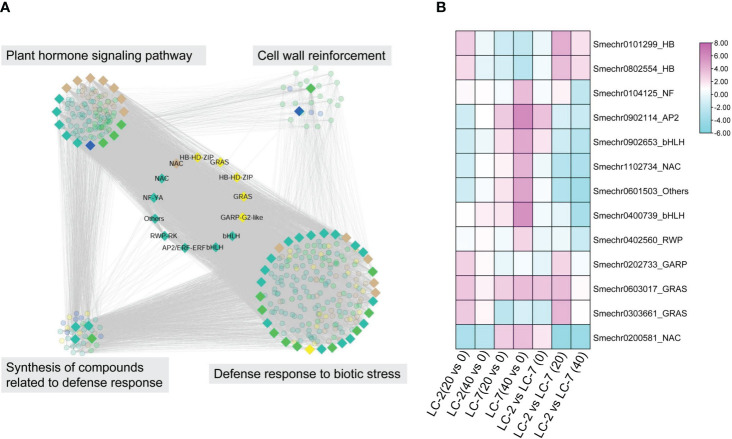
Characterization of thirteen key hub transcription factors. **(A)** In the sub-network consisting of key modules, 13 key transcription factors were co-expressed with genes involved in pathways including plant hormone signaling, defense response to biotic stress, and synthesis of compounds related to defense response. They were also indirectly co-expressed with cell wall reinforcement pathway genes through network neighbors. **(B)** Differential expression patterns (log2 fold change) of the 13 key transcription factors The first four columns represent comparisons within the strain, using the 0-day samples of LC-2 and LC-7 as controls, while the last three columns represent comparisons between strains, using LC-7 as the control. The color saturation indicates the degree of change in fold change values.

Module hub genes were identified by selecting nodes with the top 10% degree in each module. A total of 332 hub genes were screened in the five key modules (**Supplementary Table S7**), including 13 transcription factors (TFs) from the AP2, Bhlh, GRAS, NAC, NF-YA, RWP-RK, HB-HD-ZIP, GARP-G2-like, and other TF families (**Table 2**). KEGG pathway annotation revealed that some of these TFs were involved in plant–pathogen interactions, plant hormone transduction, and other pathways related to pathogen resistance. Additionally, there were some unannotated transcription factors whose functions and roles in biological stress required further research and verification. These 13 key transcription factors were co-expressed with genes of hormone response pathways, defense response pathways, and defense-related metabolite synthesis. Furthermore, their neighboring genes in the network were co-expressed with cell wall thickening genes (**Figure 7A**). The analysis of the expression patterns of these key TFs revealed that some had different response patterns during *V. dahliae* infection between LC-2 and LC-7. For example, the transcription factors Smechr0101299 and Smechr0802554 of the HB-HD-ZIP family, and Smechr0303661 of the GRAS family were induced in expression in the LC-2 after 20 days of *V. dahliae* infection treatment, while they were continuously downregulated in expression in LC-7 (**Figure 7B**). The differences in these key transcription factors between LC-2 and LC-7 may be one of the important reasons for the difference in disease resistance.

**Table 2:** Transcription factors that act as hub genes in key modules.

ID	KOG	TF-Family	module	Degree	KEGG pathway	Function
**Smechr0902114**	At3g16770	AP2/ERF-ERF	turquoise	1194	NA	Regulates resistance to Botrytis cinerea (Li et al., 2022b)
**Smechr0902653**	At1g05800_2	bHLH	turquoise	1266	ko04626: Plant-pathogen interaction	Jasmonic acid biosynthetic process and response to wounding (Wang et al., 2018)
**Smechr0400739**	At4g00050	bHLH	turquoise	1208	ko04075: Plant hormone signal transduction	Response to jasmonic acid (Zhao et al., 2021)
**Smechr0202733**	At1g68670	GARP-G2-like	yellow	309	ko04075: Plant hormone signal transduction	NA
**Smechr0603017**	At1g07530	GRAS	yellow	490	ko04075: Plant hormone signal transduction	Jasmone-induced resistance (Matthes et al., 2010)
**Smechr0303661**	At3g13840	GRAS	yellow	308	ko04075: Plant hormone signal transduction	NA
**Smechr0101299**	At1g16860	HB-HD-ZIP	yellow	450	NA	NA
**Smechr0802554**	At2g46680	HB-HD-ZIP	yellow	379	NA	NA
**Smechr1102734**	At5g63790	NAC	turquoise	1239	NA	NA
**Smechr0200581**	At2g17040	NAC	tan	607	NA	NA
**Smechr0104125**	At5g06510	NF-YA	turquoise	1226	NA	NA
**Smechr0601503**	At3g23150	Others	turquoise	1244	ko04016: MAPK signaling pathway	Involved in pathogen response (O'Donnell et al., 2003)
**Smechr0402560**	At1g20640	RWP-RK	turquoise	1191	ko04075: Plant hormone signal transduction	NA

### Correlations of module genes and metabolites

**Figure 8:**
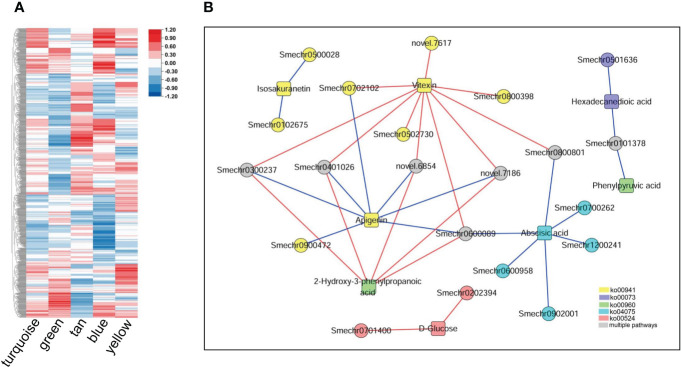
Metabolites and their associations with key modules and genes within the modules. **(A)** Correlations between metabolites detected in the metabolome and the five key modules. The heatmap shows Pearson correlation coefficients. **(B)** Genes in the key modules were significantly associated with related metabolites and participated in the same pathways. Circles represent genes; squares represent metabolites, and different colors represent different pathways: yellow represents flavonoid biosynthesis (ko00941), blue represents cutin, suberine, and wax biosynthesis (ko00073); green represents tropane, piperidine, and pyridine alkaloid biosynthesis (ko00960); cyan represents plant hormone signal transduction (ko04075), red represents neomycin, kanamycin and gentamicin biosynthesis (ko00524), and gray represents involvement in two or more of the above pathways. The red line indicates a significant positive correlation, while the blue line indicates a significant negative correlation.

The correlation analysis revealed that 236065 DEG–DAM pairs were significantly correlated, whereas only 460 metabolites were significantly correlated with the ME of the five key modules. By combining these two results, we identified 261 metabolites significantly associated with 1190 genes in key modules, as well as with the MEs of the key modules (**Figure 8A**). We further screened the metabolites and related genes to check whether they participated in the same biological pathways. This led to the identification of 35 pairs of gene–metabolite combinations in the key modules, where metabolites and related genes were involved in the same processes related to defense response. These processes included flavonoid biosynthesis, tropane, piperidine and pyridine alkaloid biosynthesis, biosynthesis of various antibiotics, cutin, suberine and wax biosynthesis, plant hormone signal transduction, neomycin, kanamycin, and gentamicin biosynthesis (**Figure 8B**). Additional information regarding these 35 gene–metabolite combinations in the key modules is provided in **Supplementary Table S8**.

### Confirmation of the transcriptome data using RT−qPCR

**Figure 9:**
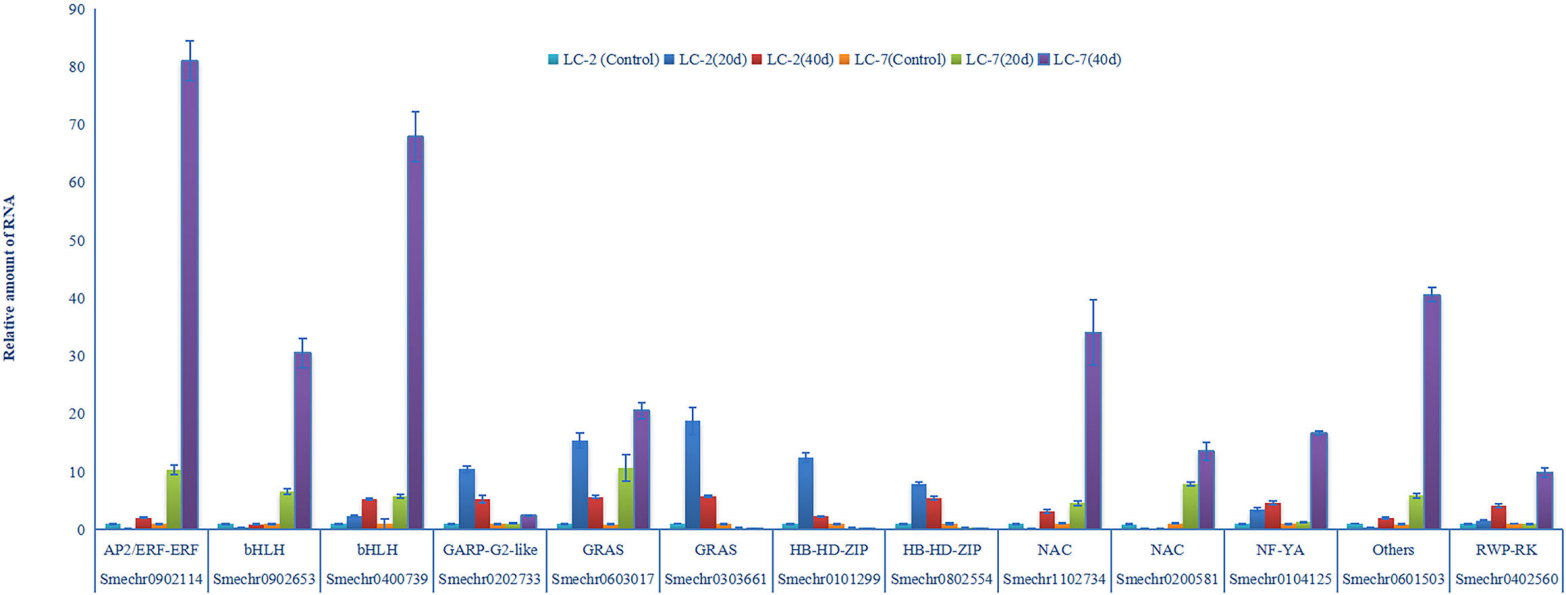
V*. dahliae*-response patterns of the 13 transcription factors in LC-2 and LC-7. The unstressed expression level (Control: uninoculated) was assigned a value of 1. The error bar on each column represents the standard deviation (SD) of the three biological replicates (Student’s *t*-test). 20 d: 20 d after inoculation, 40 d: 40 d after inoculation.

Expression levels of 13 genes were validated using RT-qPCR assays. The results of RT-qPCR assays were consistent with the transcriptome analysis, and demonstrated the accuracy of RNAseq in this study. Notably, three of the 13 transcription factors (Smechr0303661 Smechr0101299, and Smechr0802554) were induced in LC-2 and suppressed in LC-7 (**Figure 9**). These results indicated that these three genes play potentially important roles in disease resistance to *V. dahliae*.

## Discussion

In recent years, eggplant production in China has become increasingly industrialized, and continuous cropping has led to increasingly severe eggplant wilt disease, causing serious losses in eggplant production (Pisuttu et al., 2020). The research on the resistance mechanism of verticillium wilt has made breakthroughs in crops such as cotton (Gao et al., 2011), tomato (Chai et al., 2003; Fu et al., 2016), and potato (Yan et al., 2022), but little is known about the mechanism of resistance to verticillium wilt in eggplant. The rapid development of molecular biology technology has provided a foundation for the in-depth exploration of the mechanism of resistance to wilt disease in eggplant. Wild eggplant resources show excellent resistance to verticillium wilt. There are abundant species of wild eggplant in Yunnan, China. Our research group had collected more than 40 kinds of wild eggplant in Yunnan Province. The genetic relationship of these resources and their resistance to vegetable soil-borne diseases were identified (unpublished). Two eggplant species LC-2 and LC-7 from Lancang County, Puer City, Yunnan Province showed different resistance to verticillium wilt and closer genetic relationship compared with other eggplant resources. Therefore, LC-2 and LC-7 were selected as experimental materials for the combined transcriptome and metabolome analysis.

Plants activate defense mechanisms to protect themselves when attacked by pathogens, responding quickly with either direct or indirect reactions. The degree of curling of plant leaf epidermis (Singkaravanit-Ogawa et al., 2021), thickness of stratum corneum (Kempel et al., 2011) and structure of cell wall (Bacete et al., 2018), can serve as physical barriers for defense. Plants may attempt to limit the invasion and spread of pathogens by curling. In our study, a significant difference in the degree of leaf curling between LC-2 and LC-7 was observed (**Figure 1**), indicating potential differences in their defense mechanisms against diseases. The defense of eggplant not only rely on physical defense but also on innate defense mechanisms like antioxidative systems. When plants are infected with pathogens, they produce reactive oxygen species. Previous studies have shown that the accumulation of reactive oxygen species is closely related to plant disease resistance (Gayoso et al., 2010; Xu et al., 2011a). However, high levels of reactive oxygen species can have adverse effects on plant growth, and thus the expression levels of various antioxidant enzymes such as SOD, POD, and CAT will also change accordingly to protect plants from oxidative damage (Asadi et al., 2017; Huang et al., 2019). The enhanced activities of antioxidant enzymes can effectively scavenge harmful reactive oxygen species to maintain the stability of plant defense systems (Mittler et al., 2022). In tomato seedlings, the enzyme activities of the defense-related antioxidants CAT, SOD, POD, and PAL significantly increased upon inoculation with *V. dahliae* compared to controls (Pei et al., 2022). The activities of POD and PAL in cotton increased due to the infection of the V991 strain (Xu et al., 2011a). There were positive correlations between POD, PAL, and PPO activities and resistance to verticillium wilt in eggplant caused by *V. dahliae* (Zhou, 2012). In this study, we found that the six indexes of PPO, SOD, POD, PAL, GUN, and CHT in LC-2 and LC-7 showed gradually increasing trends with time after inoculation with *V. dahliae.* Furthermore, the values of the six indexes in LC-2 were higher than those in LC-7 under normal growth conditions and inoculation with *V. dahliae* (**Table 1**). This was similar to a previous research report (Zhou, 2012).

Cultivated crop wild relatives provide valuable genetic resources for crop improvement (Bohra et al., 2022). LC-2, as a wild relative of cultivated eggplant, may possess verticillium wilt resistance as a result of the combined action of multiple resistance-associated genes, given that plant disease resistance itself is a complex phenotype. Identifying genes that potentially play a role in its resistance is one of the key objectives of this study. Genetic background differences can manifest as differences in gene content, including variations in copy number and sequence. However, regardless of the form, the action of disease-resistant genes must be realized through expression in response to *V. dahliae* infection.

From a transcriptomic standpoint, LC-2 and LC-7 exhibit notable differences in gene expression in response to *V. dahliae* infection. While both strains possess a similar number of genes associated with fungal/pathogen defense mechanisms, LC-2 demonstrates a significant portion of DEGs responding to *V. dahliae* infection at an early stage (20d), whereas LC-7 exhibits a higher concentration of DEGs in the later stages of infection (40d) (**Figure 4A**). This suggests that LC-2 initiates a quicker response to *V. dahliae* infection, establishing the groundwork for its heightened resistance. Additionally, LC-2 displays a greater enrichment of genes involved in tissue development compared to LC-7 (**Figure 4B**), indicating a lesser impact of *V. dahliae* infection on the growth and development processes of LC-2. Moreover, genes associated with cell wall biogenesis and organization, serving as a dynamic barrier against pathogen invasion (Underwood, 2012), are more abundant in LC-2 than in LC-7. These findings indirectly support the superior verticillium wilt resistance observed in LC-2.

Numerous studies have indicated that co-regulated genes are often functionally related, and simultaneously recruited in co-expression network modules participating in various biological processes (van Noort et al., 2003; Romero-Campero et al., 2013; Li et al., 2022a). Here, we observed that the majority of disease resistance pathway genes are concentrated within 5 gene co-expression network modules, which are interconnected in terms of network topology and exhibit transitional functional patterns (**Figure 6**). Functional analysis revealed that these modules are involved in plant hormone signal transduction, activation of defense response genes and stress-responsive genes, cell wall biogenesis and thickening, and the synthesis of various compounds. This suggests that the response of eggplant to *V. dahliae* is the result of the integrated action of multiple processes, consistent with previous transcriptome analyses of cotton response to *V. dahliae* infection (Zhang et al., 2020; Xiong et al., 2021; Feng et al., 2023). Additionally, correlation analysis of the metabolome and transcriptome revealed associations between metabolites and co-expression network modules. We detected 261 metabolites that are simultaneously associated with module eigengenes and genes within key modules, involving multiple disease resistance-related pathways. Some metabolites have been shown to play roles in plant disease resistance, such as flavonoids, isoquinoline alkaloids, and cutin (Serrano et al., 2014; Zhang et al., 2022; Zhuang et al., 2023). Significant associations were observed between module genes and metabolites from the same pathway. These results indicate the reliability of the disease resistance-related modules obtained through gene co-expression network analysis.

The analysis of gene expression in plant-pathogen interaction pathways revealed significant expression differences in many disease resistance-related genes between LC-2 and LC-7. These differences reflect variations in the direct responses of the two strains to *V. dahliae* infection. For example, pathogenesis-related protein 1 (PR1) is known to be closely associated with plant pathogen resistance (Han et al., 2023). Among the five transcripts annotated as PR1, only one exhibits a similar differential expression pattern between LC-2 and LC-7 (**Figure 5**). Additionally, several members of the WRKY transcription factor family (including WRKY29, WRKY33, etc.) exhibit differential expression patterns between varieties. Numerous studies have demonstrated the crucial roles of WRKY family transcription factors in plant immunity and defense signaling processes (Eulgem and Somssich, 2007; Pandey and Somssich, 2009; Wani et al., 2021). The differential expression of these disease resistance-related genes between LC-2 and LC-7 likely contributes to the observed disparity in verticillium wilt resistance between LC-2 and LC-7.

Furthermore, the co-expression network analysis also revealed 13 key TFs that play potentially important roles in resistance (**Table 2**). These TFs belong to various families including AP2/ERF, bHLH, GRAS, NAC, NF-YA, RWP-RK, HB-HD-ZIP, GARP-G2-like, and others. Serving as hub genes within the network modules, they exhibit co-expression with genes involved in plant hormone signaling pathways, defense compound biosynthesis, and defense responses (**Figure 7**), implying potential associations of these TFs with various biological processes underlying disease resistance. Notably, the roles of some TFs in verticillium wilt resistance have been validated. For instance, an AP2/ERF gene, GhTINY2, is strongly induced by *V. dahliae* in cotton, directly activating WRKY51 expression and promoting salicylic acid accumulation and signal transduction, thereby enhancing resistance to *V. dahliae* (Xiao et al., 2021). In this study, the AP2/ERF gene ERF72 (SMECHR0902114) serves as the hub gene of the turquoise module, and its homolog has been demonstrated in Arabidopsis to act as a positive regulator mediating resistance to the necrotrophic pathogen Botrytis cinerea (Li et al., 2022b). Similarly, GhPAS1, a bHLH TF, is highly upregulated in cotton roots in response to *V. dahliae* invasion, and overexpression of GhPAS1 improves resistance to *V. dahliae* (Zhang et al., 2023). Two bHLH TF (SMECHR0902653 and SMECHR0400739) act in jasmonic acid were also identified as potential resistance genes in this study. Furthermore, the GRAS TF SCL14 (SMECHR0603017) were proved to play a key role in cis-jasmone induced indirect defense (Matthes et al., 2010). These findings underscore the effectiveness of our data mining approach. Furthermore, the roles of some TFs, such as the two HB-HD-ZIP family TFs Smechr0101299 and Smechr0802554, in disease resistance processes remain unclear. These transcription factors exhibit significant expression differences between LC-2 and LC-7 in response to V. dahliae. For instance, theSmechr0101299 is induced and upregulated in LC-2 upon V. dahliae infection at 20 days, whereas it remains downregulated in LC-7 (**Figure 7B**). Such differential expression patterns may contribute to the observed differences in disease resistance. Further investigation through methods such as transgenic approaches is needed to elucidate their roles in eggplant verticillium wilt resistance.

## Conclusion

In short, our study has characterized the transcriptome and metabolome of *V. dahliae* in eggplant varieties with different degrees of resistance. *V. dahliae* infection affected the enzyme activities associated with disease resistance and altered the gene expression patterns and metabolite contents. The verticillium wilt-resistant variety LC-2 exhibits higher activities of resistance-related enzymes compared to the sensitive variety LC-7. The identified DEGs and DAMs were primarily enriched in defense response, antibiotic metabolic response processes, sterol biosynthetic process, cell wall organization, plant–pathogen interaction pathways, and the disease resistance-related pathway in plant hormone signal transduction. We further identified 13 key transcription factors using a co-expression analysis. These results provide valuable insights into the molecular mechanism of eggplant resistance to verticillium wilt.

## Data availability statement

The data presented in the study are deposited in the SRA database of NCBI repository, accession number SRR28557906-SRR28557923.

## Author contributions

GL: Methodology, Writing – original draft. YM: Methodology, Writing – original draft. JL: Methodology, Writing – original draft. SH: Formal analysis, Writing – original draft. WF: Methodology, Writing – original draft. YZ: Formal analysis, Writing – original draft. ZY: Formal analysis, Writing – original draft. MD: Supervision, Writing – original draft. BX: Supervision, Writing – review & editing. YW: Supervision, Writing – review & editing. KZ: Supervision, Writing – review & editing.

## References

[B1] Asadi N. Bahmani M. Kheradmand A. Rafieian-Kopaei M. (2017). The impact of oxidative stress on testicular function and the role of antioxidants in improving it: A Review. J. Clin. Diagn. Res. 11, 1–5. doi: 10.7860/JCDR/2017/23927.9886 PMC5483704 28658802

[B2] Bacete L. Mélida H. Miedes E. Molina A. (2018). Plant cell wall-mediated immunity: cell wall changes trigger disease resistance responses. Plant J. 93, 614–636. doi: 10.1111/tpj.13807 29266460

[B3] Bohra A. Kilian B. Sivasankar S. Caccamo M. Mba C. McCouch S. R.. (2022). Reap the crop wild relatives for breeding future crops. Trends Biotechnol. 40, 412–431. doi: 10.1016/j.tibtech.2021.08.009 34629170

[B4] Chai Y. R. Zhao L. X. Liao Z. H. Sun X. F. Zuo K. J. Zhang L.. (2003). Molecular cloning of a potential *Verticillium dahliae* resistance gene SlVe 1 with multi-site polyadenylation from *Solanum licopersicoides* . DNA Seq 14, 375–384. doi: 10.1080/10425170310001605509 14756424

[B5] Cheng H. Q. Han L. B. Yang C. L. Wu X. M. Zhong N. Q. Wu J. H.. (2016). The cotton MYB108 forms a positive feedback regulation loop with CML11 and participates in the defense response against *Verticillium dahliae* infection. J. Exp. Bot. 67, 1935–1950. doi: 10.1093/jxb/erw016 26873979 PMC4783372

[B6] Dubery I. A. Slater V. (1997). Induced defence responses in cotton leaf discs by elicitors from *Verticillium dahliae* . Phytochemistry 44, 1429–1434. doi: 10.1016/S0031-9422(96)00635-8

[B7] Eulgem T. Somssich I. E. (2007). Networks of WRKY transcription factors in defense signaling. Curr. Opin. Plant Biol. 10, 366–371. doi: 10.1016/j.pbi.2007.04.020 17644023

[B8] Feng Z. L. Wei F. Feng H. J. Zhang Y. L. Zhao L. H. Zhou J. L.. (2023). Transcriptome analysis reveals the defense mechanism of cotton against *Verticillium dahliae* induced by hypovirulent fungus Gibellulopsis nigrescens CEF08111. Int. J. Mol. Sci. 24, 1480. doi: 10.3390/ijms24021480 36674996 PMC9863408

[B9] Fradin E. F. Thomma B. P. (2006). Physiology and molecular aspects of Verticillium wilt diseases caused by V. dahliae and *V. albo-atrum* . Mol. Plant Pathol. 7, 71–86. doi: 10.1111/j.1364-3703.2006.00323.x 20507429

[B10] Fu X. P. Li C. X. Zhou X. G. Liu S. W. Wu F. Z. (2016). Physiological response and sulfur metabolism of the V. dahliae-infected tomato plants in tomato/potato onion companion cropping. Sci. Rep. 6, 36445. doi: 10.1038/srep36445 27808257 PMC5093433

[B11] Gao X. Q. Wheeler T. Li Z. H. Kenerley C. M. He P. Shan L. B. (2011). Silencing GhNDR1 and GhMKK2 compromises cotton resistance to Verticillium wilt. Plant J. 66, 293–305. doi: 10.1111/j.1365-313X.2011.04491.x 21219508 PMC3078967

[B12] Gayoso C. Pomar F. Novo-Uzal E. Merino F. De Ilárduya O. M. (2010). The *Ve*-mediated resistance response of the tomato to *Verticillium dahliae* involves H2O2, peroxidase and lignins and drives PAL gene expression. BMC Plant Biol. 10, 1–19. doi: 10.1186/1471-2229-10-232 20977727 PMC3095318

[B13] Guo X. L. Shakeel M. Wang D. L. Qu C. P. Yang S. M. Ahmad S.. (2022). Metabolome and transcriptome profiling unveil the mechanisms of light-induced anthocyanin synthesis in rabbiteye blueberry (*vaccinium ashei*: Reade). BMC Plant Biol. 22, 1–14. doi: 10.1186/s12870-022-03585-x 35488209 PMC9052483

[B14] Han Z. Xiong D. Schneiter R. Tian C. (2023). The function of plant PR1 and other members of the CAP protein superfamily in plant–pathogen interactions. Mol. Plant Pathol. 24, 651–668. doi: 10.1111/mpp.13320 36932700 PMC10189770

[B15] He X. Zhu L. F. Xu L. Guo W. F. Zhang X. L. (2016). GhATAF1, a NAC transcription factor, confers abiotic and biotic stress responses by regulating phytohormonal signaling networks. Plant Cell Rep. 35, 2167–2179. doi: 10.1007/s00299-016-2027-6 27432176

[B16] Hu Q. Ao C. W. Wang X. R. Wu Y. F. Du X. Z. (2021). GhWRKY1-like, a WRKY transcription factor, mediates drought tolerance in *Arabidopsis via* modulating ABA biosynthesis. BMC Plant Biol. 21, 1–13. doi: 10.1186/s12870-021-03238-5 34625048 PMC8501554

[B17] Huang H. L. Ullah F. Zhou D. X. Yi M. Zhao Y. (2019). Mechanisms of ROS regulation of plant development and stress responses. Front. Plant Sci. 10, 800. doi: 10.3389/fpls.2019.00800 31293607 PMC6603150

[B18] Jue D. W. Yang L. Shi C. Chen M. Yang Q. (2014). Cloning and characterization of a *Solanum torvum NPR1* gene involved in regulating plant resistance to *Verticillium dahliae.* Acta Physiol. Plant 36, 2999–3011. doi: 10.1007/s11738-014-1671-0

[B19] Kawchuk L. M. Hachey J. Lynch D. R. Kulcsar F. van Rooijen G. Waterer D. R.. (2001). Tomato *Ve* disease resistance genes encode cell surface-like receptors. Proc. Natl. Acad. Sci. U. S. A. 98, 6511–6515. doi: 10.1073/pnas.091114198 11331751 PMC33499

[B20] Kempel A. Schädler M. Chrobock T. Fischer M. van Kleunen M. (2011). Tradeoffs associated with constitutive and induced plant resistance against herbivory. Proc. Natl. Acad. Sci. U. S. A. 108, 5685–5689. doi: 10.1073/pnas.1016508108 21389269 PMC3078369

[B21] Klosterman S. J. Atallah Z. K. Vallad G. E. Subbarao K. V. (2009). Diversity, pathogenicity, and management of Verticillium species. Annu. Rev. Phytopathol. 47, 39–62. doi: 10.1146/annurev-phyto-080508-081748 19385730

[B22] Kruijt M. De Kock M. J. De Wit P. J. (2005). Receptor-like proteins involved in plant disease resistance. Mol. Plant Pathol. 6, 85–97. doi: 10.1111/j.1364-3703.2004.00264.x 20565641

[B23] Lai M. J. Cheng Z. Xiao L. Y. Klosterman S. J. Wang Y. L. (2022). The bZIP transcription factor VdMRTF1 is a negative regulator of melanin biosynthesis and virulence in *Verticillium dahliae.* Microbiol. Spectr. 10, e0258121. doi: 10.1128/spectrum.02581-21 PMC9045294 35404080

[B24] Li G. Y. Zhao Y. W. Liu F. Shi M. N. Guan Y. B. Zhang T. C.. (2022a). Transcriptional memory of gene expression across generations participates in transgenerational plasticity of field pennycress in response to cadmium stress. Front. Plant Sci. 13, 953794. doi: 10.3389/fpls.2022.953794 36247570 PMC9561902

[B25] Li J. Q. Tian J. Cao H. Pu M. L. Zhang X. X. Yu Y. J.. (2023). VdMKK1-mediated cell wall integrity is essential for virulence in vascular wilt pathogen *Verticillium dahliae.* J. Genet. Genomics 50, 620–623. doi: 10.1016/j.jgg.2023.03.001 36898608

[B26] Li Y. Liu K. Tong G. Xi C. Liu J. Zhao H.. (2022b). MPK3/MPK6-mediated phosphorylation of ERF72 positively regulates resistance to *Botrytis cinerea* through directly and indirectly activating the transcription of camalexin biosynthesis enzymes. J. Exp. Bot. 73, 413–428. doi: 10.1093/jxb/erab415 34499162

[B27] Li Y. B. Han L. B. Wang H. Y. Zhang J. Sun S. T. Feng D. Q.. (2016). The thioredoxin GbNRX1 plays a crucial role in homeostasis of apoplastic reactive oxygen species in response to *Verticillium dahliae* infection in cotton. Plant Physiol. 170, 2392–2406. doi: 10.1104/pp.15.01930 26869704 PMC4825149

[B28] Liu H. P. Fu D. Q. Zhu B. Z. Yan H. X. Shen X. Y. Zuo J. H.. (2012). Virus-induced gene silencing in eggplant (*Solanum melongena*). J. Integr. Plant Biol. 54, 422–429. doi: 10.1111/j.1744-7909.2012.01102.x 22268843

[B29] Matthes M. C. Bruce T. J. Ton J. Verrier P. J. Pickett J. A. Napier J. A. (2010). The transcriptome of cis-jasmone-induced resistance in *Arabidopsis thaliana* and its role in indirect defence. Planta. 232, 1163–1180. doi: 10.1007/s00425-010-1244-4 20711606

[B30] Mittler R. Zandalinas S. I. Fichman Y. Van Breusegem F. (2022). Reactive oxygen species signalling in plant stress responses. Nat. Rev. Mol. Cell Biol. 23, 663–679. doi: 10.1038/s41580-022-00499-2 35760900

[B31] O'Donnell P. J. Schmelz E. A. Moussatche P. Lund S. T. Jones J. B. Klee H. J. (2003). Susceptible to intolerance-a range of hormonal actions in a susceptible *Arabidopsis* pathogen response. Plant J. 33, 245–257. doi: 10.1046/j.1365-313X.2003.01619.x 12535339

[B32] Pandey S. P. Somssich I. E. (2009). The role of WRKY transcription factors in plant immunity. Plant Physiol. 150, 1648–1655. doi: 10.1104/pp.109.138990 19420325 PMC2719123

[B33] Pei D. L. Zhang Q. C. Zhu X. Q. Zhang L. (2022). Biological control of Verticillium Wilt and growth promotion in tomato by rhizospheric soil-derived bacillus amyloliquefaciens Oj-2.16. Pathogens 12, 37. doi: 10.3390/pathogens12010037 36678385 PMC9865522

[B34] Pisuttu C. Pellegrini E. Cotrozzi L. Nali C. Lorenzini G. (2020). Ecophysiological and biochemical events associated with the challenge of *Verticillium dahliae* to eggplant. Eur. J. Plant Pathol. 158, 879–894. doi: 10.1007/s10658-020-02122-6

[B35] Romero-Campero F. J. Lucas-Reina E. Said F. E. Romero J. M. Valverde F. (2013). A contribution to the study of plant development evolution based on gene co-expression networks. Front. Plant Sci. 4, 54658. doi: 10.3389/fpls.2013.00291 PMC3732916 23935602

[B36] Serrano M. Coluccia F. Torres M. L’Haridon F. Métraux J. P. (2014). The cuticle and plant defense to pathogens. Front. Plant Sci. 5, 274. doi: 10.3389/fpls.2014.00274 24982666 PMC4056637

[B37] Singkaravanit-Ogawa S. Kosaka A. Kitakura S. Uchida K. Nishiuchi T. Ono E.. (2021). Arabidopsis CURLY LEAF functions in leaf immunity against fungal pathogens by concomitantly repressing SEPALLATA3 and activating ORA59. Plant J. 108, 1005–1019. doi: 10.1111/tpj.15488 34506685

[B38] Sink K. Grey W. E. (1999). A root-injection method to assess Verticillium wilt resistance of peppermint (Mentha×piperita L) and its use in identifying resistant somaclones of cv black Mitcham. Euphytica 106, 223–230. doi: 10.1023/A:1003591908308

[B39] Song R. R. Li J. P. Xie C. J. Jian W. Yang X. R. (2020). An overview of the molecular genetics of plant resistance to the Verticillium Wilt pathogen *Verticillium dahliae* . Int. J. Mol. Sci. 21, 1120. doi: 10.3390/ijms21031120 32046212 PMC7037454

[B40] Su X. F. Lu G. Q. Guo H. M. Zhang K. X. Li X. K. Cheng H. M. (2018). The dynamic transcriptome and metabolomics profiling in *Verticillium dahliae* inoculated *Arabidopsis* thaliana. Sci. Rep. 8, 15404. doi: 10.1038/s41598-018-33743-x 30337674 PMC6193927

[B41] Tomah A. A. Alamer I. S. A. Khattak A. A. Ahmed T. Hatamleh A. A. Al-Dosary M. A.. (2023). Potential of *Trichoderma virens* HZA14 in controlling *Verticillium* Wilt disease of eggplant and analysis of its genes responsible for microsclerotial degradation. Plants (Basel). 12, 3761. doi: 10.3390/plants12213761 37960117 PMC10649075

[B42] Tzima A. Paplomatas E. J. Rauyaree P. Kand S. (2010). Roles of the catalytic subunit of cAMP dependent protein kinasee a in virulence and development of the soil-brone plant pathogen *Verticillium dahliae.* Fungal Genet. Biol. 47, 406–415. doi: 10.1016/j.fgb.2010.01.007 20144723

[B43] Underwood W. (2012). The plant cell wall: a dynamic barrier against pathogen invasion. Front. Plant Sci. 3, 25639. doi: 10.3389/fpls.2012.00085 PMC3355688 22639669

[B44] van Noort V. Snel B. Huynen M. A. (2003). Predicting gene function by conserved co-expression. Trends Genet. 19, 238–242. doi: 10.1016/S0168-9525(03)00056-8 12711213

[B45] Wang K. Guo Q. Froehlich J. E. Hersh H. L. Zienkiewicz A. Howe G. A.. (2018). Two abscisic acid-responsive plastid lipase genes involved in jasmonic acid biosynthesis in *Arabidopsis thaliana* . Plant Cell. 30, 1006–1022. doi: 10.1105/tpc.18.00250 29666162 PMC6002186

[B46] Wani S. H. Anand S. Singh B. Bohra A. Joshi R. (2021). WRKY transcription factors and plant defense responses: latest discoveries and future prospects. Plant Cell Rep. 40, 1071–1085. doi: 10.1007/s00299-021-02691-8 33860345

[B47] Xiao S. H. Hu Q. Zhang X. J. Si H. Liu S. M. Chen L.. (2021). Orchestration of plant development and defense by indirect crosstalk of salicylic acid and brassinosteorid signaling via transcription factor GhTINY2. J. Exp. Bot. 72, 4721–4743. doi: 10.1093/jxb/erab186 33928361

[B48] Xiong X. P. Sun S. C. Zhu Q. H. Zhang X. Y. Liu F. Li Y. J.. (2021). Transcriptome analysis and RNA interference reveal *GhGDH2* regulating cotton resistance to Verticillium Wilt by JA and SA signaling pathways. Front. Plant Sci. 12, 654676. doi: 10.3389/fpls.2021.654676 34177978 PMC8226099

[B49] Xu L. Zhu L. F. Tu L. L. Guo X. P. Long L. Sun L. Q.. (2011a). Differential gene expression in cotton defence response to *Verticillium dahliae* by SSH. J. phytopathology. 159, 606–615. doi: 10.1111/j.1439-0434.2011.01813.x

[B50] Xu L. Zhu L. F. Tu L. L. Liu L. L. Yuan D. J. Jin L.. (2011b). Lignin metabolism has a central role in the resistance of cotton to the wilt fungus *Verticillium dahliae* as revealed by RNA-Seq-dependent transcriptional analysis and histochemistry. J. Exp. Bot. 62, 5607–5621. doi: 10.1093/jxb/err245 21862479 PMC3223054

[B51] Yan N. N. Addrah M. E. Zhang Y. Y. Jia R. F. Kang L. R. Zhao J.. (2022). Identification and comparison of biological characteristics and pathogenicity of different mating types of V. dahliae isolated from potato and sunflower. Sci. Rep. 12, 12840. doi: 10.1038/s41598-022-17196-x 35896720 PMC9329468

[B52] Yang L. Mu X. Y. Liu C. Cai J. H. Shi K. Zhu W. J.. (2015). Overexpression of potato miR482e enhanced plant sensitivity to *Verticillium dahliae* infection. J. Integr. Plant Biol. 57, 1078–1088. doi: 10.1111/jipb.12348 25735453

[B53] Yang J. Zhang Y. Wang X. F. Wang W. Q. Li Z. K. Wu J. H.. (2018). HyPRP1 performs a role in negatively regulating cotton resistance to *Verticillium dahliae via* the thickening of cell walls and ROS accumulation. BMC Plant Boil. 18, 1–18. doi: 10.1186/s12870-018-1565-1 PMC6286592 30526498

[B54] Zhang X. Y. Cheng W. H. Feng Z. D. Zhu Q. H. Sun Y. Q. Li Y. J.. (2020). Transcriptomic analysis of gene expression of *Verticillium dahliae* upon treatment of the cotton root exudates. BMC Genom. 21, 1–25. doi: 10.1186/s12864-020-6448-9 PMC7017574 32050898

[B55] Zhang J. Guo M. Z. Wu H. H. (2023). *GhPAS1*, a bHLH transcription factor in upland cotton (*Gossypium hirsutum*), positively regulates *Verticillium dahliae* resistance. Ind. Crops Prod. 192, 116077. doi: 10.1016/j.indcrop.2022.116077

[B56] Zhang C. Ou X. Wang J. Wang Z. Du W. Zhao J.. (2022). Antifungal peptide P852 controls Fusarium wilt in faba bean (Vicia faba L.) by promoting antioxidant defense and isoquinoline alkaloid, betaine, and arginine biosyntheses. Antioxidants 11, 1767. doi: 10.3390/antiox11091767 36139841 PMC9495604

[B57] Zhao K. Shen X. J. Yuan H. Z. Liu Y. Liao X. Wang Q.. (2013). Isolation and characterization of dehydration-responsive element-binding factor 2C (MsDREB2C) from Malus sieversii Roem. Plant Cell Physiol. 54, 1415–1430. doi: 10.1093/pcp/pct087 23757363

[B58] Zhao P. Z. Zhang X. Gong Y. Q. Wang D. Xu D. Q. Wang N.. (2021). Red-light is an environmental effector for mutualism between begomovirus and its vector whitefly. PloS Pathog. 17, e1008770. doi: 10.1371/journal.ppat.1008770 33428670 PMC7822537

[B59] Zhou B. L. (2012). Correlation between resistance of eggplant and defense-related enzymes and biochemical substances of leaves. Afr J. BioMed. Res. 11, 13896–13902. doi: 10.5897/AJB

[B60] Zhou X. H. Liu J. Zhuang. Y. (2014). Selection of appropriate reference genes in eggplant for quantitative gene expression studies under different experimental conditions. Sci. Hortic. 176, 200–207. doi: 10.1016/j.scienta.2014.07.010

[B61] Zhuang W. B. Li Y. H. Shu X. C. Pu Y. T. Wang X. J. Wang T.. (2023). The classification, molecular structure and biological biosynthesis of flavonoids, and their roles in biotic and abiotic stresses. Molecules 28, 3599. doi: 10.3390/molecules28083599 37110833 PMC10147097

[B62] Zuo K. J. Qin J. Zhao J. Y. Ling H. Zhang L. D. Cao Y. F.. (2007). Over-expression GbERF2 transcription factor in tobacco enhances brown spots disease resistance by activating expression of downstream genes. Gene 391, 80–90. doi: 10.1016/j.gene.2006.12.019 17321073

[B63] Zuo K. Wang J. Wu W. Chai Y. Sun X. Tang K. (2005). Identification and characterization of differentially expressed ESTs of Gossypium barbadense infected by *Verticillium dahliae* with suppression subtractive hybridization. Mol. Biol. (Mosk). 39, 214–223. doi: 10.1007/s11008-005-0028-6 15856944

