# Supplementary figures and images for: Unraveling verticillium wilt resistance: insight from the integration of transcriptome and metabolome in wild eggplant
Source: Front Plant Sci. 2024 May 28;15:1378748. doi: 10.3389/fpls.2024.1378748 (PMC11165189; doi:10.3389/fpls.2024.1378748)

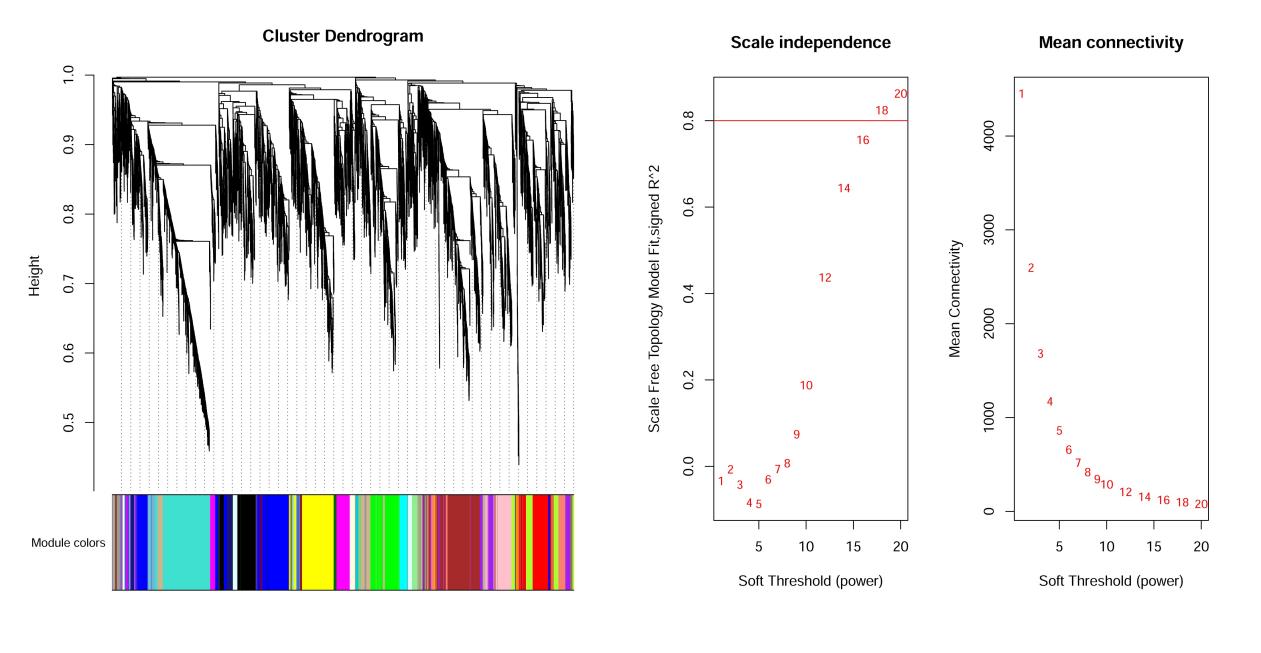


Supplementary Figure S1 Gene co-expression network construction

Supplement: Supplementary file 1 [file DataSheet_1.docx]
