# Supplementary material for: Unraveling verticillium wilt resistance: insight from the integration of transcriptome and metabolome in wild eggplant
Source: Front Plant Sci. 2024 May 28;15:1378748. doi: 10.3389/fpls.2024.1378748 (PMC11165189; doi:10.3389/fpls.2024.1378748)
Supplement: Supplementary file 2 [file DataSheet_2.docx]

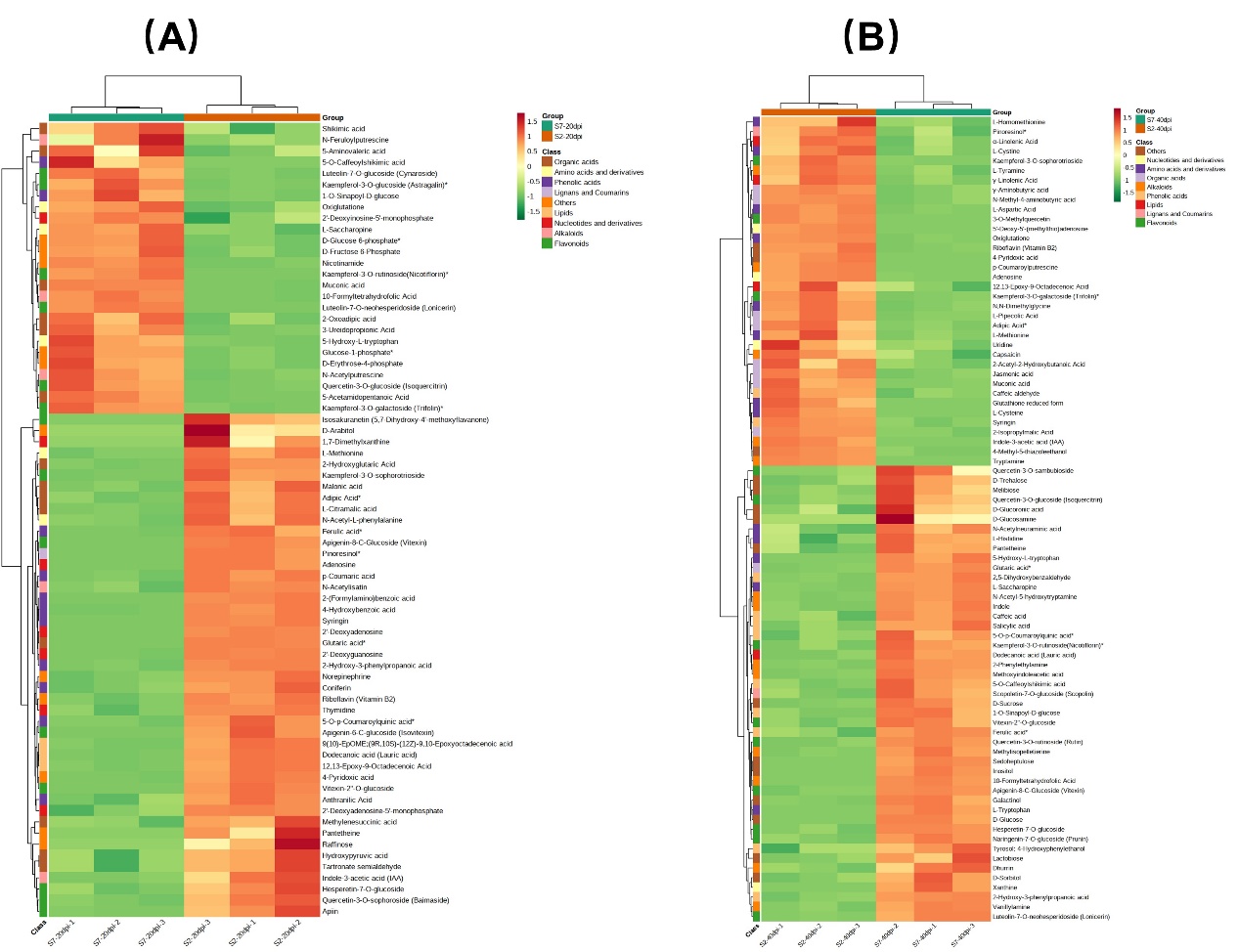


Supplementary Figure S2. Heatmap of Major classes of detected metabolites of DAMs of LC-2 vs. LC-7 at 20 days (A) and 40 days (B). Three independent replicates of each stage are displayed in the heat map.
