# Supplementary material for: Unraveling verticillium wilt resistance: insight from the integration of transcriptome and metabolome in wild eggplant
Source: Front Plant Sci. 2024 May 28;15:1378748. doi: 10.3389/fpls.2024.1378748 (PMC11165189; doi:10.3389/fpls.2024.1378748)
Supplement: Supplementary file 3 [file DataSheet_3.docx]

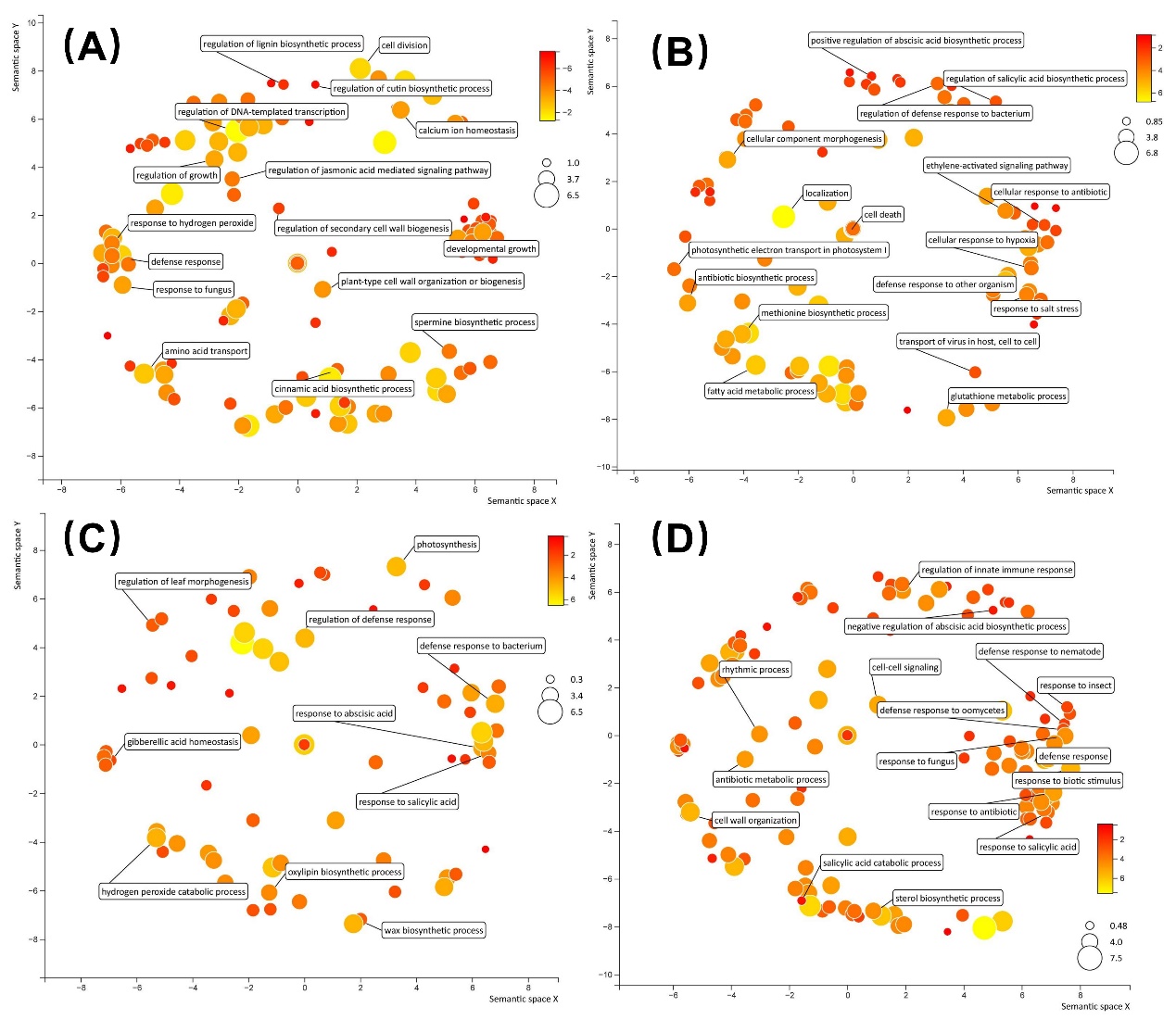


Supplementary Figure S3. Scatterplot of biological processes associated with genes induced by *V. dahliae* in LC-2 and LC-7. (A) LC-2 specific DEGs. (B) LC-7 specific DEGs. (C) DEGs that showed similar expression patterns between LC-2 and LC-7. (D) DEGs that showed differential expression patterns between LC-2 and LC-7. The color and size are proportional to the p-value and log10 p-value of GO enrichment analysis, respectively.
