# Supplementary material for: Unraveling verticillium wilt resistance: insight from the integration of transcriptome and metabolome in wild eggplant
Source: Front Plant Sci. 2024 May 28;15:1378748. doi: 10.3389/fpls.2024.1378748 (PMC11165189; doi:10.3389/fpls.2024.1378748)
Supplement: Supplementary file 4 [file DataSheet_4.docx]

Supplementary Table S1 Selected candidate reference genes and primers for the quantitative real-time PCR

| Genes | Forward primer (5’-3’) | Reverse primer (5’-3’) |
| --- | --- | --- |
| GAPDH | CCGCTCCTAGCAAAGATGCC | ACCCTCCACAATGCCAAACC |
| Smechr0902114 | GTCCATGGGGTAAATGGGCA | AATGTTACGGGGAGGCTCTG |
| Smechr0902653 | TCACACCTTGGTCCTTCACG | TGCCACCAGTTTGTGTGGAT |
| Smechr0400739 | TGTTAACATGCCCCCTTCCC | TGGTAAAACGCTGCCATCCT |
| Smechr0202733 | TTCAGGTTCAGCCAAAGGGG | AGCCTGATTTCACAAACAAACA |
| Smechr0603017 | CATCCGCTTCAGAATCCCTCA | TCTGATGGTCGTCTGCTTGA |
| Smechr0303661 | GCTTAGCTGGGGGTTCATGTT | CTATGGTGGAAAGGACAGCCA |
| Smechr0101299 | ACGCAGGGGAATTTTCTCCAA | TGGCTCAAGTTTGGTCTCCA |
| Smechr0802554 | ACAGAACCATTTTGTCAGTTGAGT | GCCTGAACTTTCCCCCAGTT |
| Smechr1102734 | CAAACTAAGCAAGCAGCAGCA | GTCGTCGGTCGGATGGAATC |
| Smechr0200581 | GGCACCTAAAGGCACCAGAA | CATGTCGATCGGCTGACCAT |
| Smechr0104125 | TCTTACACCACGCAAGCCAT | TGAATGTTGCCGGTCTTGGA |
| Smechr0601503 | TGCCACTTGAAATTTGGGTGA | TGGCCTATCTGTGTGCAAGAA |
| Smechr0402560 | TGGGACAAAGCCACATTCTCA | TCTCAGCGACAGATGCATGG |
