# Supplementary material for: Unraveling verticillium wilt resistance: insight from the integration of transcriptome and metabolome in wild eggplant
Source: Front Plant Sci. 2024 May 28;15:1378748. doi: 10.3389/fpls.2024.1378748 (PMC11165189; doi:10.3389/fpls.2024.1378748)
Supplement: Supplementary file 5 [file DataSheet_5.docx]

Supplementary Table S2 Part agronomic traits of LC-2 and LC-7

| Varieties | Plant type | Main stem color | Plant height(cm) | Stem dimeter  (cm) | Main stem prickle | Leaf shape | Leaf color | Leaf thorn | Leaf length (cm) | Blade width  (cm) | Corolla color | Weight of single fruit (g) | Color of ripe fruit | Fruit shape | Edge groove on fruit surface |
| --- | --- | --- | --- | --- | --- | --- | --- | --- | --- | --- | --- | --- | --- | --- | --- |
| LC-2 | upright | purple | 83 | 1.6 | yes | Long oval | green | small amount | 16.2 | 12.8 | white | 112.5 | orange | oblate | yes |
| LC-7 | upright | green | 131 | 2.0 | no | Long oval | green | none | 19.6 | 15.5 | purple | 1.6 | orange | round | none |
