# Supplementary material for: Unraveling verticillium wilt resistance: insight from the integration of transcriptome and metabolome in wild eggplant
Source: Front Plant Sci. 2024 May 28;15:1378748. doi: 10.3389/fpls.2024.1378748 (PMC11165189; doi:10.3389/fpls.2024.1378748)
Supplement: Supplementary file 7 [file DataSheet_7.docx]

Supplementary Table S4 Information and quality control of transcriptome sequencing

| Sample | Raw Reads | Clean Reads | Clean Base(G) | Error Rate(%) | Q20(%) | Q30(%) | GC Content(%) |
| --- | --- | --- | --- | --- | --- | --- | --- |
| Sample | Raw Reads | Clean Reads | Clean Base(G) | Error Rate(%) | Q20(%) | Q30(%) | GC Content(%) |
| LC-2-0dpi-1 | 50783312 | 49711564 | 7.46 | 0.03 | 97.52 | 93 | 43.83 |
| LC-2-0dpi-2 | 49763926 | 48778658 | 7.32 | 0.03 | 97.48 | 92.88 | 42.79 |
| LC-2-0dpi-3 | 50362568 | 49470560 | 7.42 | 0.03 | 97.42 | 92.77 | 43.02 |
| LC-7-0dpi-1 | 61028692 | 56891440 | 8.53 | 0.03 | 97.56 | 93.08 | 41.37 |
| LC-7-0dpi-2 | 41378032 | 40009678 | 6 | 0.03 | 96.98 | 91.83 | 41.58 |
| LC-7-0dpi-3 | 55641184 | 45591514 | 6.84 | 0.03 | 96.5 | 91.64 | 40.18 |
| LC-2-20dpi-1 | 47771782 | 44591950 | 6.69 | 0.03 | 97.89 | 93.79 | 46.54 |
| LC-2-20dpi-2 | 41825348 | 40112230 | 6.02 | 0.03 | 98.02 | 94.03 | 43.5 |
| LC-2-20dpi-3 | 47607680 | 45947604 | 6.89 | 0.03 | 96.94 | 91.68 | 43.58 |
| LC-2-40dpi-1 | 42106704 | 40766714 | 6.12 | 0.03 | 96.98 | 91.82 | 42.13 |
| LC-2-40dpi-2 | 39811084 | 38787114 | 5.82 | 0.03 | 97.22 | 92.32 | 43.33 |
| LC-2-40dpi-3 | 44984288 | 43332414 | 6.5 | 0.03 | 97.28 | 92.47 | 43.08 |
| LC-7-20dpi-1 | 46709662 | 45125216 | 6.77 | 0.03 | 97.12 | 92.17 | 44.25 |
| LC-7-20dpi-2 | 41238126 | 39660926 | 5.95 | 0.03 | 97.05 | 91.97 | 44.72 |
| LC-7-20dpi-3 | 41489474 | 40026554 | 6 | 0.03 | 97.27 | 92.49 | 44.81 |
| LC-7-40dpi-1 | 45617928 | 44605002 | 6.69 | 0.03 | 97.95 | 93.79 | 43.68 |
| LC-7-40dpi-2 | 52421290 | 50587552 | 7.59 | 0.03 | 97.27 | 92.42 | 42.73 |
| LC-7-40dpi-3 | 47160566 | 45059042 | 6.76 | 0.03 | 97.29 | 92.46 | 42.43 |
