# Supplementary material for: Unraveling verticillium wilt resistance: insight from the integration of transcriptome and metabolome in wild eggplant
Source: Front Plant Sci. 2024 May 28;15:1378748. doi: 10.3389/fpls.2024.1378748 (PMC11165189; doi:10.3389/fpls.2024.1378748)
Supplement: Supplementary file 8 [file DataSheet_8.docx]

Supplementary Table S5 Statistics of regional distribution of transcripts

| Sample | Exon (%) | Intron (%) | Intergenic (%) |
| --- | --- | --- | --- |
| LC-2-0dpi-1 | 85.42 | 6.29 | 8.29 |
| LC-2-0dpi-2 | 87.55 | 5.2 | 7.25 |
| LC-2-0dpi-3 | 86.85 | 5.55 | 7.6 |
| LC-2-20dpi-1 | 89.78 | 3.9 | 6.32 |
| LC-2-20dpi-2 | 89.64 | 3.98 | 6.37 |
| LC-2-20dpi-3 | 89.68 | 3.99 | 6.33 |
| LC-2-40dpi-1 | 87.9 | 4.87 | 7.24 |
| LC-2-40dpi-2 | 88.69 | 4.6 | 6.71 |
| LC-2-40dpi-3 | 88.29 | 4.76 | 6.94 |
| LC-7-0dpi-1 | 85.68 | 5.87 | 8.45 |
| LC-7-0dpi-2 | 87.04 | 5.25 | 7.71 |
| LC-7-0dpi-3 | 83.83 | 6.31 | 9.87 |
| LC-7-20dpi-1 | 87.09 | 5.36 | 7.55 |
| LC-7-20dpi-2 | 86.53 | 5.64 | 7.83 |
| LC-7-20dpi-3 | 87.39 | 5.26 | 7.35 |
| LC-7-40dpi-1 | 89.72 | 4 | 6.28 |
| LC-7-40dpi-2 | 88.72 | 4.3 | 6.98 |
| LC-7-40dpi-3 | 87.85 | 4.79 | 7.35 |
